# A Mathematical Model of Mitotic Exit in Budding Yeast: The Role of Polo Kinase

**DOI:** 10.1371/journal.pone.0030810

**Published:** 2012-02-23

**Authors:** Baris Hancioglu, John J. Tyson

**Affiliations:** Department of Biological Sciences, Virginia Polytechnic Institute and State University, Blacksburg, Virginia, United States of America; National Cancer Institute, United States of America

## Abstract

Cell cycle progression in eukaryotes is regulated by periodic activation and inactivation of a family of cyclin–dependent kinases (Cdk's). Entry into mitosis requires phosphorylation of many proteins targeted by mitotic Cdk, and exit from mitosis requires proteolysis of mitotic cyclins and dephosphorylation of their targeted proteins. Mitotic exit in budding yeast is known to involve the interplay of mitotic kinases (Cdk and Polo kinases) and phosphatases (Cdc55/PP2A and Cdc14), as well as the action of the anaphase promoting complex (APC) in degrading specific proteins in anaphase and telophase. To understand the intricacies of this mechanism, we propose a mathematical model for the molecular events during mitotic exit in budding yeast. The model captures the dynamics of this network in wild-type yeast cells and 110 mutant strains. The model clarifies the roles of Polo-like kinase (Cdc5) in the Cdc14 early anaphase release pathway and in the G-protein regulated mitotic exit network.

## Introduction

The cell cycle plays a crucial role in all biological growth, reproduction and development, and the molecular machinery underlying the cell cycle is known to be highly conserved among all eukaryotes [1]. Faithful transmission of genetic information depends on accurate chromosome segregation as cells exit from mitosis, and the penalty for errors in chromosome segregation is severe; failures in this process lead to aneuploidy which is responsible for many cases of spontaneous abortions, birth defects and cancer [2]. In eukaryotes, an elaborate molecular control system ensures the proper orchestration of events at mitotic exit (ME). Understanding how cell division is controlled by this network of interacting genes and proteins is clearly important to the life sciences, the biotech industry and medical science.

**Figure 1:**
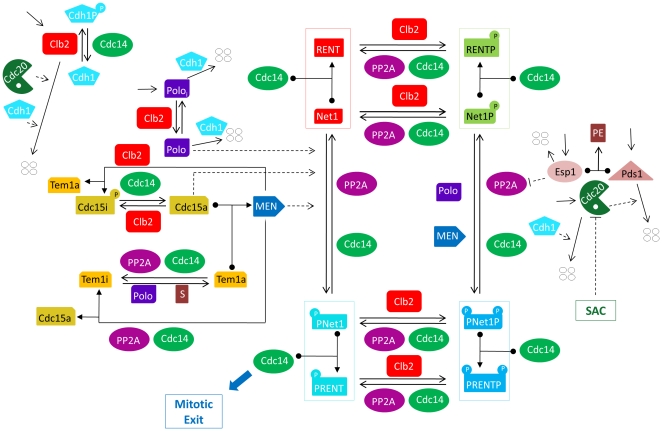
Proposed wiring diagram of mitotic exit control in the budding yeast cell cycle. For a full justification of this diagram with references, see Texts S1 and S2. Cdc28, the kinase partner of Clb2, is not shown explicitly in this diagram. Cdc20 and Cdh1 work in collaboration with the APC, which is also not shown explicitly in the diagram. All proteins (ovals) are assumed to be produced and degraded at specific rates. Four white circles represent degraded proteins. Solid lines correspond to chemical reactions, while dashed lines denote regulatory effects (enzyme catalysis). A protein sitting on a reaction arrow also represents an enzyme that catalyses the reaction. Cdc20 initiates the transition from metaphase to anaphase. Cdc14 released from PRENT and PRENTP induces exit from mitosis, i.e., activation of Cdh1 and establishment of the cell in G1 phase.

The molecular events during ME are particularly well delineated in budding yeast, *Saccharomyces cerevisiae*, for which a large collection of well characterized ME-mutant strains are available. From the phenotypes of these mutants, yeast geneticists are able to propose a hypothetical network of interactions among the proteins encoded by ME genes. However, the resulting network (e.g., Figure 1) is so complex that it defies understanding by intuitive reasoning alone. As an aid to intuition, we propose a mathematical model of the ME control system. We show that the model is consistent with the observed phenotypes of most ME-mutants in budding yeast, and we use the model to predict the behavior of the ME network under novel conditions. This methodology has been used to advantage for many years to create mathematical models of cell cycle regulation in fission yeast [3]–[8], budding yeast [9], and mammalian cells [10]. Embryonic cell cycles have been modeled in frog eggs [11], the fruit fly [12] and the sea urchin [13]. Not only have these models reproduced large amounts of experimental data, but also they have made successful predictions and guided further experimental studies [14]–[16].

Since 2004, when Chen *et al.* [9] published their comprehensive model of the budding yeast cell cycle, many more molecular details of ME have come to light, and several updated models of ME have been proposed [17]–[19]. In this paper, we present a mathematical model of ME control, taking into account the essential role that Polo kinase (Cdc5) plays in the phosphorylation of Net1 and the subsequent release of Cdc14 from the nucleolus [20]. We propose a novel mechanism for phosphorylation of Net1 on distinct sites by the ME-relevant kinases: Cdc28, Cdc5 and Dbf2/Mob1 (through activation by Cdc15). The model also integrates proteolytic and nonproteolytic functions of Esp1 into the Cdc14 early anaphase release (FEAR) pathway and the mitotic exit network (MEN). The model accounts for the observed properties of ME in wild-type yeast cells and 110 mutant strains, and it predicts the phenotypes of numerous mutant yeast cells that have not yet been studied to our knowledge.

The precise molecular mechanism by which Cdc5 promotes Net1 phosphorylation, FEAR activation, and ME is not known. Cdc5 promotes FEAR activation in part by inducing degradation of Swe1 (an inhibitor of Cdk/Clb2 activity), which enables Cdk/Clb2 to phosphorylate Net1 [21]. Cdc5 reduces the affinity between Net1 and Cdc14 [22]. Cdc5 phosphorylates Net1 extensively *in vitro*, and it may influence the phosphorylation state of Net1 *in vivo* [23]–[25].

Our model of ME is based on well-known biochemical interactions in budding yeast and on the assumption that Cdc5 phosphorylates Net1 *in vivo* on its own. In our model, dissociation of Cdc14 from Net1 relies on Net1 being phosphorylated solely by Cdc5 or being multiply phosphorylated by Cdk/Clb2, by MEN and by Cdc5. In this paper, we gather all the evidence supporting Net1 phosphorylation by Cdc5 *in vivo* on its own, using observed phenotypes of mutant yeast cells to clarify the mechanism of Cdc14 activation during ME.

The exact role of Polo kinase (Cdc5) in the ME process and the exact mechanism by which Net1 gets phosphorylated and Cdc14 is released are the most controversial aspects of ME. Our view that Net1 can be solely phosphorylated by Cdc5 has been challenged by others [21]. Recent models of ME consider Net1 phosphorylation to be dependent on Cdk and MEN-kinases [17], [19]. In Queralt's model [17], Cdc5 cannot phosphorylate Net1 on its own, and the essential role of Cdc5 in ME is attributed to its role in MEN. Later on, Vinod *et al.* [19] extended Queralt's model with more cell cycle regulators, including Net1 phosphorylation by Cdc5. However, Vinod's model assumes that Net1 phosphorylation by Cdc5 is dependent on a priming phosphorylation by Cdk/Clb2 or MEN kinases. At the heart of our model, unique to this paper, lies the assumption that Cdc5 may phosphorylate Net1 on its own, independent of Cdk and MEN phosphorylation.

For further contextualization, we refer readers to Text S1, where we summarize some details of ME kinetics in budding yeast and the interactions among major components of the control system. In the next section, we provide details about how these interactions are implemented in our mathematical model.

## Results

### A model for mitotic exit in budding yeast

Our proposed mechanism for ME in yeast (Figure 1) is simplified by combining the roles of some of the cell cycle proteins described in Text S1. For instance, we represent the two G1-stabilizers (Cdh1 and Sic1) by a single variable, with the properties of Cdh1. We combine the ‘mitotic cyclins’ (Clb1-4) into a single variable, Clb2. We assume that Cdk subunits (Cdc28) are always available to bind to Clb2, since Cdc28 is present in excess in cells. Hence, the model focuses on the synthesis and degradation of Clb2 and ignores fluctuations in Cdc28 level. Some proteins that play known roles in the metaphase-anaphase-G1 transition (ME), such as Sic1, Lte1, Bub2 and Bfa1, have been left out of the present model. We plan to include them in a later version, along with a representation of the chromosome alignment checkpoint. For a discussion of the model's assumptions, please see Text S2.

Our model focuses on the cell cycle transition from a stable metaphase state (high Cdk activity, low Cdh1 activity) to a stable G1 state (low Cdk activity and high Cdh1 activity). This transition corresponds to a window of the cell cycle that has been frequently studied experimentally by arresting cells in metaphase by Cdc20-depletion, followed by synchronous release into anaphase by readdition of Cdc20 or by overexpression of separase, e.g. [17].

In this article, we aim to clarify the factors affecting activation and inactivation of FEAR and MEN pathways, the functions of Cdc5 and Esp1 to promote ME, and the regulation of Cdc14 (release from and re-sequestration to the nucleolus) in the wild-type cell cycle. Since the publication of Queralt's model [17], new molecular details of ME, especially regarding to the roles of Cdc5 [20] have come to light. To account for the experiments in Visintin *et al.* [20] and many others, we have extended Queralt's model with some new components and interactions. Nonetheless, the consensus picture of the regulation of most ME regulators (summarized in Text S1) has not changed, so we retain from Queralt's model the same ODEs—or very similar ones—for these ME regulators (Clb2, Cdc20, Pds1, Esp1, PE, Polo, Tem1, Cdc15 and MEN). The main challenge is to find out how these regulators are wired together in ME pathways. We examined many alternative scenarios. Each scenario is consistent with many observed phenotypes but inconsistent with other important observations. It is not our purpose in this paper to discuss all these alternative scenarios but to present the one that we believe to be the best.

Unique to our model, we propose a mechanism for Cdc14 regulation by multisite phosphorylation of Net1 by several kinases, as depicted in Figure 1. We propose that Cdc5 may phosphorylate Net1 *in vivo* on its own, either before or after Net1 is phosphorylated by Cdk [23]. As opposed to the assumption of Queralt's model, which attributes the essential role of Cdc5 later in ME as part of MEN, we consider Cdc5 as an essential component of both MEN and FEAR pathways. We propose that Cdc5 can induce Cdc14 release by phosphorylating Net1 directly even when other components of FEAR and MEN pathways are silent [20]. Cdc15 acts only in the MEN pathway, downstream of Tem1 and upstream of Dbf2/Mob1, the kinase that phosphorylates Net1 [26]–[29]. When Tem1 is inactive, overexpressed Cdc15 can still activate MEN and sustain Cdc14 release [30]. To explain overexpressed Cdc15 mutants when Tem1 is inactive, our model assumes that active Cdc15 can phosphorylate Net1 on its own as well. In the Queralt model, Cdc15 is part of the MEN complex, which phosphorylates Net1, but Cdc15 cannot phosphorylate Net1 on its own.

Key regulators of ME are the protease Esp1 [31] and the phosphatase Cdc14 [32]. Active Esp1 promotes anaphase (separation of sister chromatids) by cleaving cohesin rings. Esp1 is kept inactive in early M phase by binding to a stoichiometric inhibitor, Pds1 (securin). Cdc14, when active, promotes ME by dephosphorylating the proteins that were phosphorylated by Cdk/Clb kinases in the run-up to metaphase. Cdc14 is kept inactive in early M phase by binding to a stoichiometric inhibitor Net1, found in the nucleolus. The Cdc14/Net1 complex is known as RENT (regulator of nucleolar silencing and telophase) [33], [34]. At ME, both Pds1 and Net1 must be neutralized. Pds1 is degraded by proteasomes after polyubiquitination by the Cdc20/APC complex. Net1 is inactivated by phosphorylation by FEAR and MEN. In metaphase, even though Cdk/Clb2 and Cdc5 are actively phosphorylating Net1, Net1 is kept active by a powerful phosphatase Cdc55/PP2A [17], [35], [36].

When all chromosomes are correctly aligned on the mitotic spindle, Cdc20/APC becomes active and initiates degradation of both Clb2 and Pds1. Degradation of Pds1 releases Esp1, which promotes anaphase (its catalytic activity as a protease). Esp1 has a second, non-catalytic function to inhibit PP2A, allowing Cdk/Clb2 and Cdc5 to phosphorylate Net1 and release Cdc14.

The regulation of Tem1 is more complicated than indicated in Figure 1. Tem1 is a G-protein, i.e., it is active when bound to GTP and inactive when GTP is hydrolyzed to GDP [37]. GTP hydrolysis is promoted by the Bub2/Bfa1 complex and GDP-GTP exchange is promoted by Lte1 [29], [38]. By phosphorylating and inactivating Bfa1, Cdc5 activates Tem1 [39], [40]. Contrariwise, PP2A and Cdc14 dephosphorylate Bfa1 and inactivate Tem1 [17], [20], [41]. In addition, Tem1 activation is promoted at anaphase II (spindle elongation), when Lte1 is brought into contact with Tem1 by migration of the daughter spindle pole body to the bud cortex [29]. In the present model, we simplify this mechanism by introducing Tem1i and Tem1a (inactive and active), and associating Tem1 activation to Cdc5 and “S” (spindle elongation), and Tem1 inactivation to PP2A and Cdc14.

Cdc15 activity is regulated by phosphorylation and dephosphorylation (Figure 1). Cdc15 is phosphorylated (inhibited) by Cdk/Clb2 and dephosphorylated (activated) by Cdc14. Hence, FEAR-induced Cdc14 release may promote MEN activation through Cdc15 dephosphorylation [28], [42]–[44]. The positive feedback loop between Cdc14 and Cdc15 keeps MEN active, which sustains Cdc14 release until Cdh1 is fully activated [17].

Cdh1 activity is also regulated by phosphorylation and dephosphorylation (Figure 1). Cdh1 is inactive in metaphase because it is strongly phosphorylated by Cdk/Clb2. During ME, Cdh1 is activated when the rising phosphatase activity of Cdc14 overcomes the falling kinase activities of Clb2 and Cdc5 [32], [45], [46]. Because Cdh1/APC degrades Clb2 and Cdc5, it establishes the newborn cells in G1 phase after ME.

Progression through exit from mitosis and the temporal order of late mitotic events depend, at least in part, on the order in which different Cdk and APC targets are dephosphorylated and destroyed [47]. Both Cdc14 and PP2A dephosphorylate Net1 [17], [33], [35], [36], [42]. Early dephosphorylation of Cdc15 may be a problem for the proper organization of ME events by early activation of MEN. Hence, we assume that PP2A, which is high in metaphase and in early anaphase, does not dephosphorylate Cdc15. Rather, Cdc14 activates MEN by dephosphorylating Cdc15 [28], [42]–[44], after activation of the FEAR pathway. PP2A and Cdc14 dephosphorylate Bfa1 and inactivate Tem1 [17], [20], [41]. To simplify quantitative description in the model, Tem1 is inactivated by PP2A/Cdc55 and Cdc14 rather than acting through GAP and GEF. Cdh1 is activated by Cdc14 in late anaphase by dephosphorylation when PP2A is low [46], [48], [49].

Figure 1 summarizes this introduction in the form of a hypothetical wiring diagram of the molecular interactions controlling ME in budding yeast. We use mathematical modeling to assess the adequacy of this wiring diagram to account for known facts about ME in wild-type and mutant budding yeast cells. The model also makes predictions about the phenotypes of novel mutants that have not yet been characterized experimentally, and these mutant properties can serve as independent tests of the wiring diagram. Our modeling methods are described briefly in the accompanying “Methods” section. The mathematical equations that describe the dynamics of the molecular interactions in Figure 1 are given in Table S1.

### The model describes the regulation of Cdc14 in wild-type cells

**Figure 2:**
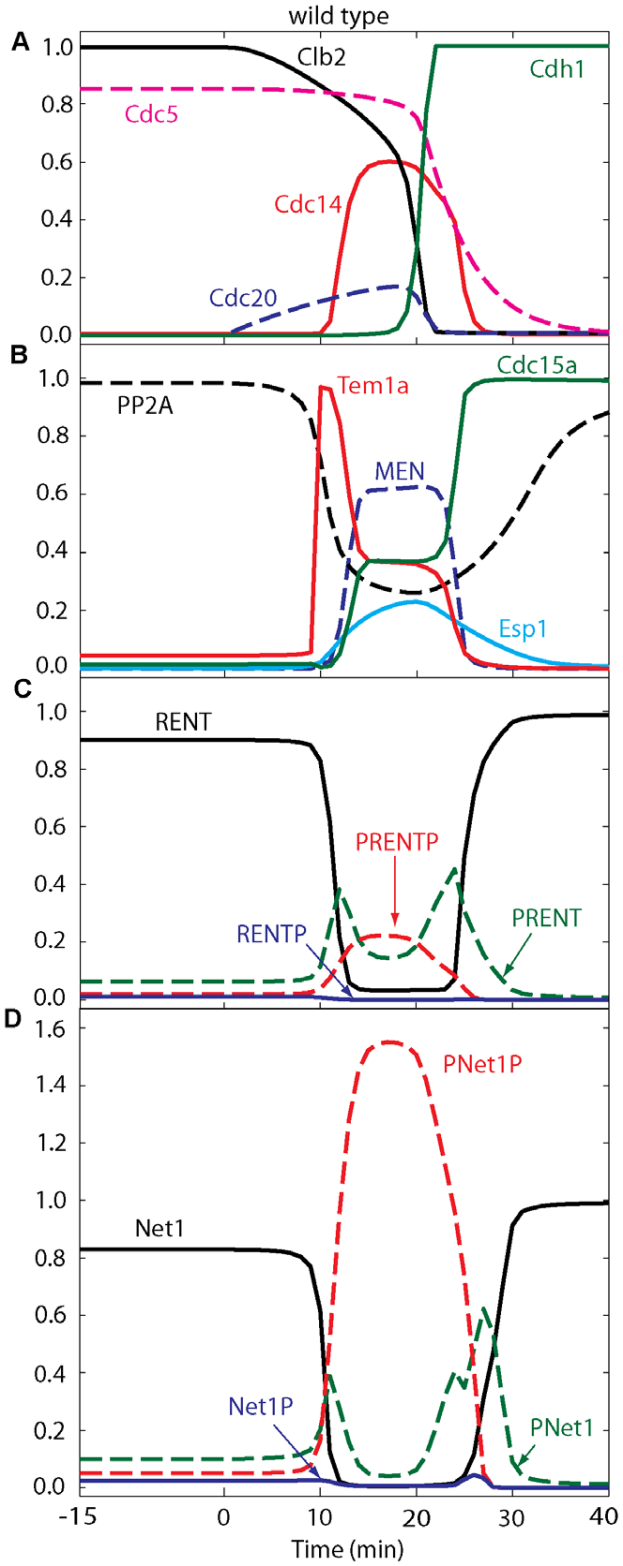
Numerical simulation of exit from mitosis in wild-type cells. The four panels show the time courses of ME regulators during a typical Cdc20 ‘block and release’ experiment, which is simulated as follows: the simulation starts at *t* = −15 min under metaphase-block conditions (*cdc20Δ GAL-CDC20* in glucose; *k*_s,20_ = 0), and then Cdc20 synthesis is induced (transfer to galactose; *k*_s,20_ = 0.015) at *t* = 0. During the Cdc20-block phase (*t*<0), free Cdc14 is low due to sequestration by Net1 in RENT, MEN is inactive, and Net1 and RENT are predominantly dephosphorylated because of high activity of PP2A. The steady state levels of Clb2, Cdc5, Net1, RENT and PP2A are close to 1 (arbitrary unit).

In Figure 2 we simulate ME events based on the differential equations in Table S1, the ‘basal’ set of rate constants for wild-type cells (Table S2), and the initial conditions (Table S3) that represent a cell arrested in metaphase. In this paper, ‘wild type’ refers to cells of the mutant strain *cdc20ΔGAL-CDC20*, which can be arrested in metaphase by Cdc20-depletion (growth on glucose) and then induced to exit mitosis by adding back Cdc20 (transferring cells from glucose to galactose medium) at *t* = 0. In Figure 2, as in all simulations, we plot the (scaled) concentrations of representative proteins: Clb2, Cdc5, Cdc14 (released), Cdh1 (active), etc. The simulation is in good agreement with experimental observations of ME in wild-type cells [17]. During metaphase arrest by Cdc20-depletion, Cdc14 is sequestered in RENT, MEN activity is negligible, and all phosphorylated forms of Net1 and RENT are small. The steady state levels of Clb2, Cdc5, Net1, RENT and PP2A are at their peak values, close to 1 (arbitrary unit).

**Figure 3:**
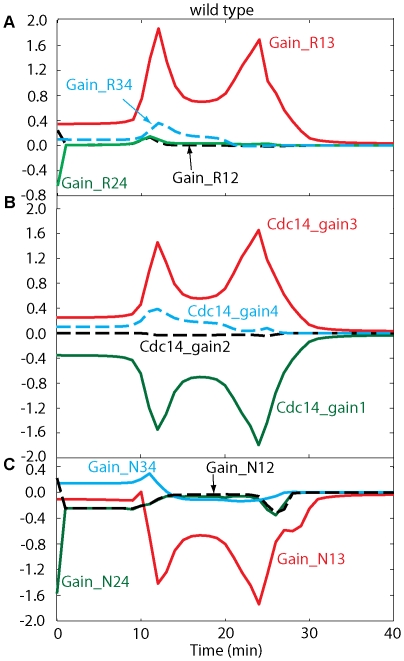
Flux diagrams in wild-type cells. Initially cells are in the metaphase steady state by Cdc20 deprivation. Cdc20 activation at time zero (*k*_s,20_ = 0.015) induces mitotic progression through anaphase, telophase and G1. Flux definitions are given in Table S1.

The metaphase-arrested state is very dynamic, with rapid rates of phosphorylation and dephosphorylation of Net1, due to high activities of kinases (Cdk/Clb2 and Cdc5) and the opposing phosphatase, PP2A (Flux diagrams are presented in Figure 3). In metaphase, Cdc5 activity is high, and it continuously phosphorylates RENT to PRENT, which rapidly dissociates into Cdc14 and PNet1. At the same time, high activity of Cdk/Clb2 phosphorylates Net1 subunits to PRENTP, which also dissociates rapidly to Cdc14 and PNet1P. Nonetheless, PP2A activity is also high in metaphase, and this phosphatase converts PNet1 and PNet1P to Net1. Under these conditions, there is an excess of Net1, which avidly captures free Cdc14, sequestering it back into RENT. The dynamic balance among these fluxes in metaphase (Figure 3) maintains high steady state levels of Net1 and RENT, and low levels of the phosphorylated forms of these proteins.

This dynamic balance is disturbed at the metaphase-anaphase transition (*t* = 0 in Figure 2) by the production of Cdc20, which has several consequences. (1) By degrading a fraction of Clb2 (∼40%), it lowers both Cdk and Cdc5 activities. (2) By degrading Pds1, it releases Esp1 to down-regulate the activity of PP2A. (3) The phosphatase activity drops much more than the kinase activities, causing net phosphorylation of Net1 subunits and release of Cdc14 from RENT complexes. (4) Cohesin cleavage by Esp1 leads to spindle elongation (*S* = 1) and activation of Tem1. Because these processes are self-reinforcing, there is an abrupt release of Cdc14 from the nucleolus about 10 min after the onset of Cdc20 synthesis (transfer to galactose medium).

Tem1 activation alone is insufficient for MEN activity; Cdc15 must be activated as well, and this requires FEAR-mediated release of Cdc14. Active MEN now contributes to phosphorylation of RENT and RENTP into PRENT and PRENTP, from which Cdc14 is further released in a sustained manner during anaphase and telophase. Cdc14 reaches its peak (0.6) around *t* = 15 min and stays at peak level for ∼10 min. Notice that in our model fluxes are much higher along the route RENT→PRENT→PRENTP than along the route RENT→RENTP→PRENTP, which suggests that Cdc5 phosphorylation may prime Net1 for phosphorylation by Cdk/Clb2, rather than *vice versa*.

After MEN activation, the fluxes from RENT to PRENT and from PNet1 to Net1 intensify; thus, PNet1 stays relatively low while PNet1P is high. Eventually the Cdc14∶Clb2 ratio becomes large enough to activate Cdh1. Active Cdh1 completes the ubiquitination and degradation of Clb2, as well as promoting the degradation of Cdc5 and Cdc20. Loss of Cdc20 results in re-accumulation of Pds1, which inhibits Esp1, allowing PP2A phosphatase activity to go back up. High PP2A activity and free Cdc14 turn PNet1P into Net1, which captures the remaining Cdc14 back into RENT. The return of Cdc14 to the nucleolus is dependent on both Cdc14 and PP2A phosphatase activities.

The binding rate of Cdc14 to Net1 is quite high and the unbinding rate is low, making RENT the most stable complex relative to the others. In RENTP, Cdc14 binding rate to Net1P is much lower than the binding to Net1 in RENT, but it is ten orders of magnitude higher than the binding in PRENT and PRENTP; in addition, the unbinding rate is low as in RENT. Hence, RENTP is a much more stable complex than either PRENT or PRENTP. Because the dissociation rates of PRENT and PRENTP are large, Cdc14 is released from PRENT and PRENTP during anaphase and telophase.

The variable *S* (spindle elongation) starts to drop after Cdh1 reaches 0.3 around 23 min, at which time we assume cytokinesis occurs. (The quantitative criterion for ME in our model is that Cdh1 increases above 0.3.) At *t* = 35 min, Cdh1 is fully active and PP2A is high, while Cdk/Clb2, Cdc5 and MEN activities are close to zero, and Cdc14 is sequestered back in the nucleolus. The system has come to the G1 steady state, where all the fluxes are negligible, Cdh1 is active, the levels of RENT, Net1, Cdc15, and PP2A are close to 1, and the concentrations of all other model variables are close to zero.

**Figure 4:**
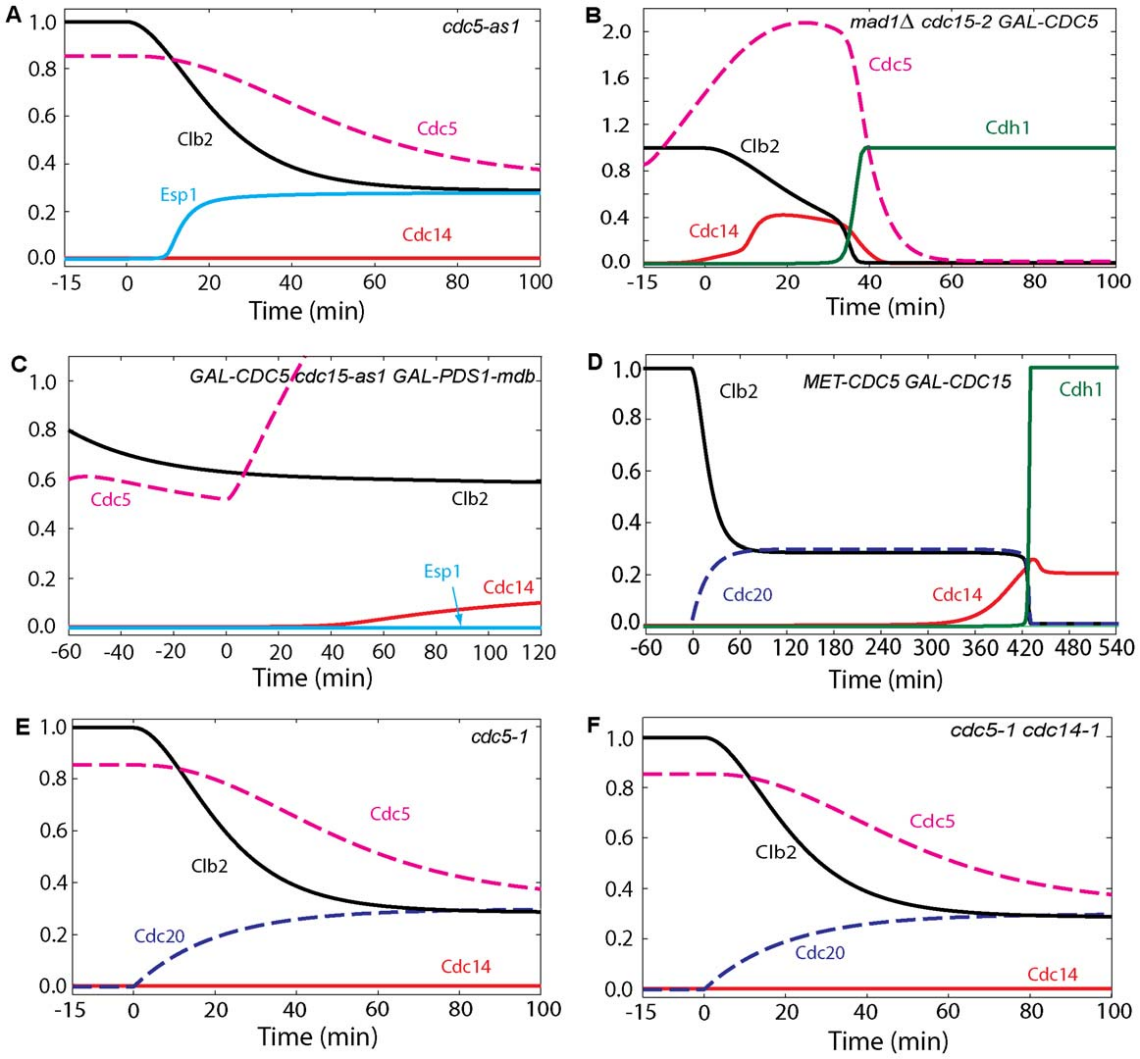
Simulation of mitotic progression of cells containing overexpressed *CDC5* and inactive *cdc5* mutations. (**A**) Cdc5 is necessary for ME. Cdc20 block-and-release was simulated as in Figure 2 with inactive Cdc5 (*cdc5-as1*; *effpol* = 0). Cdc14 is not released, nor is Cdh1 activated. (**B**) The MEN requirement for ME can be bypassed by overexpressed Cdc5. Cdc20 block-and-release was simulated as usual, with inactive Cdc15 (*cdc15-2*; *effc15* = 0) and with Cdc5 overexpressed 30-fold (*GAL-CDC5*; *k*_s,polo_ = 0.3). (**C**) Overexpressed Cdc5 is sufficient for Cdc14 release when FEAR and MEN are inactive. Simulation was started in an arrested steady state with initial conditions of Clb2 and Polo were set less than metaphase values to represent an earlier stage of the arrest by hydroxyurea (Clb2 = 0.8, Polo = 0.6, Poloi = 0.2, *k*_s,b2_ = 0.024, *k*_s,polo_ = 0.006) and with inactive Cdc15 (*effc15* = 0) for 15 min. Then Cdc5 and Pds1 overexpressions were induced at time zero (*k*_s,polo_ = 0.3, *k*_s,pds_ = 0.45, *k*_d,pds′_ = 0). (**D**) The Cdc5 requirement for Cdc14 release and ME can be bypassed by overexpression of a truncated version of Cdc15. Cdc20 block-and-release was pre-simulated for 60 min with no synthesis of either Cdc20 or Cdc5 (*k*_s,polo_ = *k*_s,20_ = 0; setting also the initial conditions for Cdc5 active and inactive forms to zero) while the total concentration of Cdc15 was increased 20-fold and inhibition of Cdc15 by Cdk was reduced 1000-fold (*k*_i,c15_′ = 0.00009, *CDC15T* = 20). At *t* = 0, Cdc20 synthesis is induced as usual (*k*_s,20_ = 0.015). (**E**) Cdc14 is not released in *cdc5-1* and *cdc5-1 cdc14-1* cells in E and F. Therefore, Cdc14 release in the *cdc14-1* mutant may be attributable solely to Net1 phosphorylation by Cdc5. Simulation in E was done similar to Figure 4A except that *effpol* was set to 0.1 for the small residual activity of Cdc5. (**F**) Simulation in F was done similar to A except that activity of Cdc14 was set to zero (*effc14* = 0).

For all our model simulations, we indicate the figure presenting the particular model simulation, such as “simulated in Figure 4D”, and the paper presenting the simulated experiment(s), with a literature citation. If any statement in the paper does not include “simulated in Figure number”, then it is either our proposal or claim (if no reference is given) or an experimental finding (if a reference is given).

The mathematical model must be consistent not only with the properties of ME in wild-type cells but also with the phenotypes of budding yeast strains that carry mutations in ME genes. To test our model, we simulate ME mutants of yeast using exactly the same differential equations, parameter values, and initial conditions as for wild-type cells (Tables S1, S2, and S3), except for those modifications to parameters dictated by the particular mutation being simulated (see model web page). We describe the most informative mutants in the text, we provide additional information in the Supporting Information, and we provide a full account of all mutant simulations on the web page that supports this paper (http://mpf.biol.vt.edu/research/mitotic_exit_model/pp).

### Cdc5 is necessary and sufficient for Cdc14 release

That Cdc5 is necessary for Cdc14 release is demonstrated by the *cdc5-as1* mutant (simulated in Figure 4A), for which Polo activity is very low, Net1 is not phosphorylated, and Cdc14 is not released [20]. Cdc14 is retained in RENT in our simulations, and the fluxes are negligible. Cdc5 has many other roles in addition to Cdc14 release in anaphase. Cdc5 may regulate microtubule function, spindle orientation, and migration [50]. *cdc5-as1* cells are arrested in telophase with short mitotic spindles [49], [51], large buds, separated DNA masses, and elevated Cdk/Clb2 activity [50]. In a majority of anaphase *cdc5-as1* cells, spindle elongation occurred entirely in the mother cell, rather than through the bud neck, which implies a failure of the nucleus to migrate into the bud [50]. Thus, Tem1 may be prevented from gaining access to Lte1 [39] and stays inactive. Since Cdc5 has significant impact on spindle elongation, Tem1 activation by S depends on Polo in our model equations.

Overexpressed Cdc5 can promote Cdc14 release and ME when both MEN and FEAR are inactive. In the mutant *GAL-CDC5 cdc15-2*, Cdc5 can be overexpressed in the absence of Cdc15 activity, allowing Visintin *et al.* [30] to show that overexpressed Cdc5 can induce Cdc14 release when MEN is inactive (simulated in Figure 4B). The *GAL-CDC5 cdc15-as1 GAL-PDS1-mdb* mutant [20] shows that overexpressed Cdc5 is sufficient to promote Cdc14 release when both MEN and FEAR are inactive, because Cdc15 is inactive and Pds1 is abundant (simulated in Figure 4C). In this figure, Clb2 is elevated due to inactive Cdc2 (In these experiments, cells are arrested in S phase by hydroxyurea, which blocks the activation of Cdc20). High Clb2-kinase activity blocks ME (activation of Cdh1), and Cdc14 is not re-sequestered to the nucleolus. When Cdc20 is active, as in *mad1Δ cdc15-2 GAL-CDC5* cells [30], ME and Cdc14 release occur with almost the same kinetics as wild-type cells (simulated in Figure 4B). Even though MEN is inactive, overexpressed Cdc5 is sufficient for Cdc14 release and ME. Cdc5 is dispensable when MEN is hyperactivated by overexpression of a truncated version of Cdc15 (see Figure 4D), which may explain the phenotype of the *met-cdc5-repress GAL-CDC15[1-750]* mutant strain [30].

In contrast to other MEN mutants (simulated in Figure 4E), Cdc14 is not transiently released in *cdc5-1* cells [52], and Net1 is in a hypophosphorylated form in telophase arrest [21] because of the greatly reduced Polo activity. Net1 is not phosphorylated in the *cdc5-1 cdc14-1* double mutant (simulated in Figure 4F); hence, Cdc14 release in *cdc14-1* may be attributable only to Net1 phosphorylation by Cdc5.

The model predicts that after Cdc20 addition *GAL-CDC5 cdc15-2 NET1-6cdk* mutant cells exit from mitosis with a delay, but *GAL-CDC5 cdc15-2 GAL-PDS1* cells are arrested in telophase (see model webpage). In *GAL-CDC5 cdc15-2 GAL-PDS1*, Cdc14 is transiently released and returns to the nucleolus because Cdk activity is reduced due to degradation of Clb2 by Cdc20, which causes Polo activity to go down. Hence, Cdc14 release can no longer be sustained.

### FEAR promotes transient Cdc14 release in early anaphase

**Figure 5:**
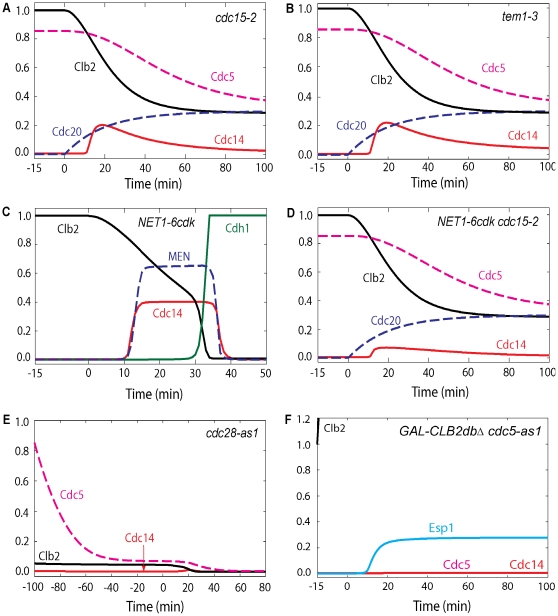
Simulations of mitotic progression of cells containing *cdc15-2*, *NET1-6cdk*, *tem1-3, cdc28-as1*, and *GAL-CLB2dbΔ cdc5-as1* mutations. (**A**) In MEN mutants such as *cdc15-2*, Cdc14 is transiently released and resequestered. Cdc20 block-and-release was presimulated with inactive Cdc15 (*effc15* = 0). (**B**) In *tem1-3* temperature sensitive mutant (MEN inactive), Cdc14 is transiently released and cells are arrested in telophase. Simulation was started at metaphase by Cdc20 deprivation (*k*_s,20_ = 0) for 15 min with total concentration of Tem1, initial conditions of Tem1 and MEN were set to zero. Cdc20 was activated at time zero (*k*_s,20_ = 0.015) (**C**) In *NET1-6cdk* cells ME occurs with a delay, as typical of FEAR mutants. Cdc20 block-and-release was presimulated with no Net1 phosphorylation by Cdk/Clb2 (*k*_k,12_ = *k*_k,34_ = 0). (**D**) Double MEN and FEAR mutations, such as *NET1-6cdk cdc15-2*, do not show transient release of Cdc14 and arrest in telophase. Cdc20 block-and-release was presimulated with inactive Cdc15 (*effc15* = 0) and no Net1 phosphorylation by Cdk/Clb2 (*k*_k,12_ = *k*_k,34_ = 0). (**E**) When Cdk kinase activity is inhibited, there is no Cdc14 release. Both Cdk/Clb2 and Cdc5 phosphorylation on Net1 are diminished in *cdc28-as1* mutant. Simulation was done similar to wild-type cells except that INH was set to 5 to inhibit Cdk kinase activity. (**F**) Our model predicts that when Cdc5 is inhibited, overexpressed Clb2 cannot induce Cdc14 release with or without active Cdc20. Simulation was started at metaphase by Cdc20 deprivation, overexpression of Clb2 and inactive Cdc5 for 15 min (*k*_s,20_ = 0, *k*_s,b2_ = 0.6, *k*_d,b2_ = *k*_d,b2′_ = effpol = 0). Cdc20 was added back at time zero (*k*_s,20_ = 0.015).

Mutants defective in MEN components, such as *cdc15-2*, *tem1-3*, and *dbf2-2*, are arrested in telophase with elongated spindles. After a transient release in early anaphase (simulated in Figure 5A and Figure 5B), Cdc14 is tightly sequestered in the nucleolus [33], [34], [52], [53]. In MEN mutants (e.g. *cdc15-2* in Figure 5A), the initial time of FEAR-induced Cdc14 release is about 11 min in our simulation, as in experiments [52]. Clb2 is degraded to almost half its mitotic level by Cdc20 [54] but no further because Cdh1 remains inactive. Cdc15 is transiently activated in *dbf2-2* mutants; however, it stays inactive in *cdc14-3* [52], confirming that FEAR-induced Cdc14 dephosphorylates and activates Cdc15, promoting its ability to activate MEN [42].

In cells carrying the *NET1-6cdk* mutation, where all Cdk phosphorylation sites on Net1 are mutated [55], ME is delayed by about 13 min, similar to other FEAR defective mutants in our simulations (simulated in Figure 5C). In *NET1-6cdk* mutant cells, Cdc5 phosphorylates Net1 and RENT after PP2A drops at anaphase onset. Cdc14 is released from PRENT only, because Net1 cannot be phosphorylated by Cdk/Clb2. This causes prolonged Cdc14 release induced by Cdc5 and MEN before ME. *NET1-6cdk* cells are considered FEAR mutants because *NET1-6cdk cdc15-2* cells [55] fail to exhibit FEAR-release of Cdc14 (simulated in Figure 5D) in the absence of Cdk/Clb2 and MEN phosphorylation on Net1.

Clb2-dependent phosphorylation of Net1 is also absent in strains carrying the *cdc28-as1* allele (encoding Cdk1 protein that can be inhibited by the drug 1NM-PP1). Chemical inhibition of Cdk1 kinase activity causes inactivation of Cdc5, which results in elimination of Net1 phosphorylation and Cdc14 release (simulated in Figure 5E) [55]. Therefore, Cdk/Clb2 phosphorylation is important for the timely exit from mitosis and contributes to FEAR-release of Cdc14; however, unlike Cdc5, Cdk1 is not playing an essential role for FEAR. Our model predicts that when Cdc5 activity is inhibited, as in *cdc5-as1 GAL-CLB2-dbΔ* cells, RENT is phosphorylated by Cdk/Clb2 to RENTP, which does not readily dissociate; thus, overexpression of Clb2 protein cannot induce Cdc14 release in the absence of Cdc5 activity (simulated in Figure 5F).

**Figure 6:**
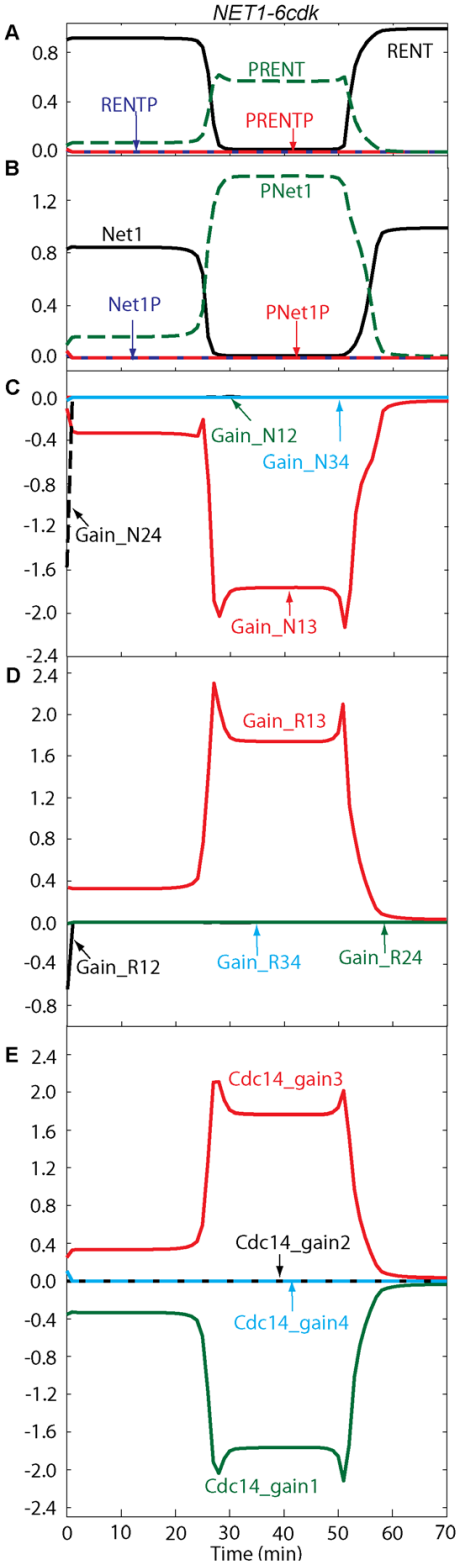
Flux diagrams and temporal changes of RENT, Net1 forms in *NET1-6cdk* mutants. Simulation was done similar to Figure 5C.

**Figure 7:**
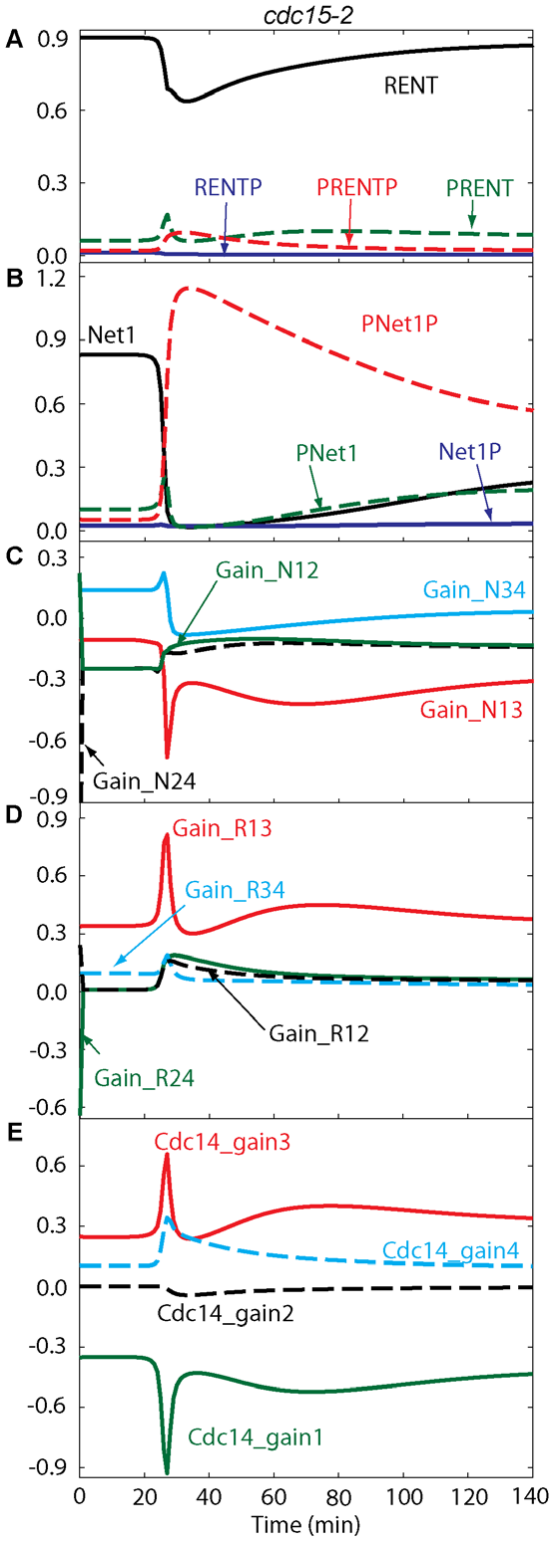
Temporal changes of RENT, Net1 forms and fluxes in *cdc15-2* cells blocked at telophase in mitosis. Simulation was done similar to Figure 5A.

The flux diagrams for *NET1-6cdk* and *cdc15-2* mutant cells are shown in Figure 6 and Figure 7, respectively. Figure 7 shows that, after anaphase onset, fluxes in the path RENT
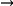
PRENT
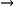
PRENTP are increased because of high Cdc5. Fluxes through RENT
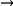
RENTP
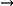
PRENTP do not play a major role in the transient release of Cdc14 because of higher phosphatase activity in this path (as in wild-type cells). In *cdc15-2* cells, Net1 gets phosphorylated first by Cdc5 and then by Cdk/Clb2 in our model, and Cdc14 gets released from PRENT and PRENTP. At telophase arrest, all variables reach their steady state values where some portion of Net1 stays in the phosphorylated form, and kinase activities are reduced to about half of their metaphase levels. Cdc14 reaches a peak value, which is less than half of its peak in wild-type cells, before it is sequestered back into RENT. RENT returns to metaphase levels, and PRENT stays a little elevated due to remaining Cdc5 activity. PNet1P and PNet1, released from PRENT and PRENTP, are dephosphorylated by Cdc14 and move back to RENT after capturing free Cdc14.

**Figure 8:**
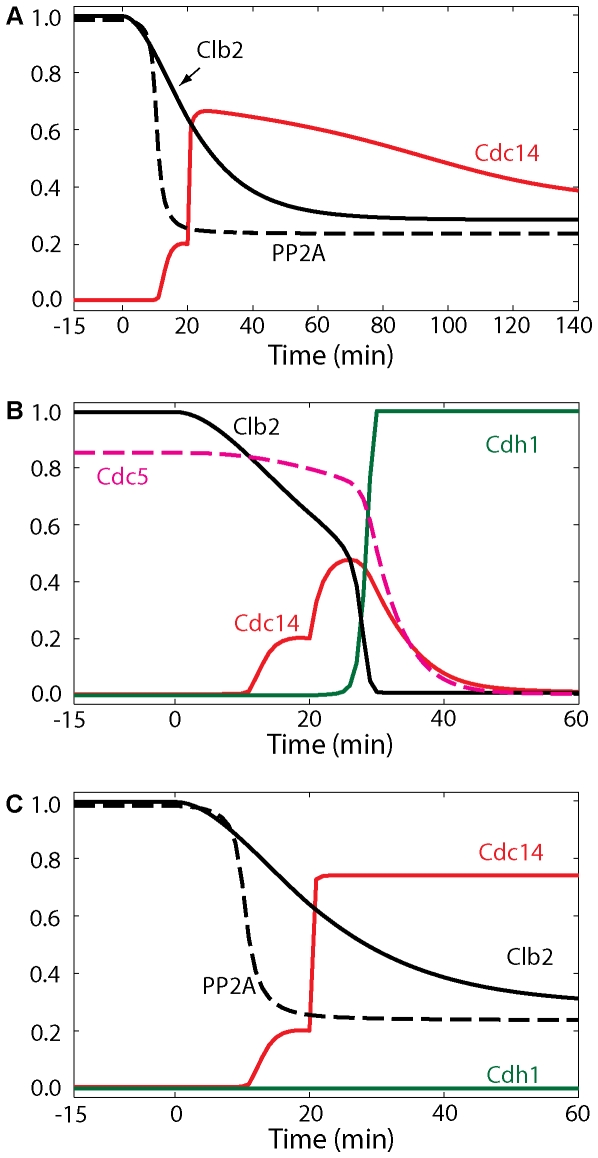
Model predicts that Cdc14 is responsible for its own re-sequestration after ME. (**A–C**) All simulations were done similar to *cdc15-2* mutant simulations in Figure 5A except that after 20 min either Cdc14 (in A, *effc14* = 0) or PP2A (in B, *effppa* = 0) or both (in C, *effc14* = *effppa* = 0)) were inactivated by setting their corresponding activity factors to zero.

The model predicts that, after Cdc14 transient release in *cdc15-2* cells, inhibition of Cdc14 phosphatase activity results in an elevated level of Cdc14 protein released in telophase (Figure 8A); however, inhibition of PP2A activity has no such effect on Cdc14 release (Figure 8B). When both Cdc14 and PP2A phosphatase activities are inhibited the effect is similar to the inhibition of Cdc14 (Figure 8C). Therefore, in our model Cdc14 itself is responsible for its own re-sequestration after its transient release in MEN mutants. FEAR-released Cdc14 in MEN mutants cannot induce ME because the Cdc14/Clb2 ratio stays below the critical threshold to activate Cdh1.

### Both proteolytic and nonproteolytic activity of Esp1 contributes to ME

**Figure 9:**
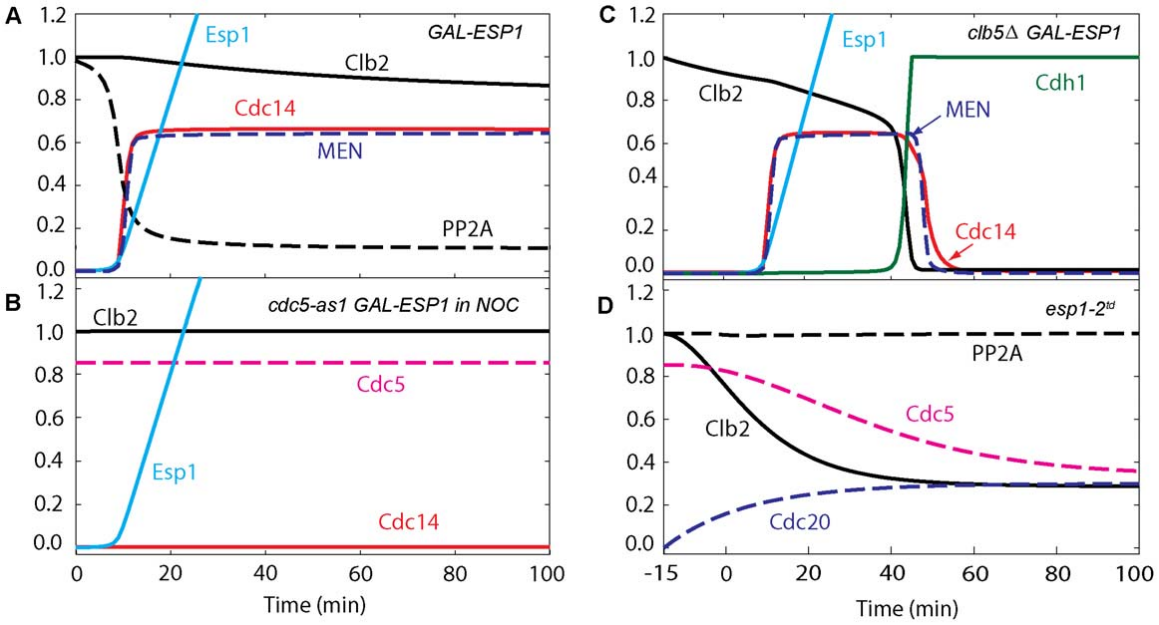
Mitotic progression of cells containing an *ESP1* mutation. (**A**) In metaphase arrested cells at 23°C, overexpression of Esp1 induces Cdc14 release; however, cells do not exit from mitosis, and Cdh1 stays inactive. Cells are presimulated in metaphase arrest by Cdc20 deprivation, then at *t* = 0 the rate of synthesis of Esp1 is increased 60-fold (*k*_s,esp_ = 0.078), with the rate of Clb2 degradation at 23°C assumed to be half its basal value (*k*_d,b2″_ = 1.5). (**B**) Cdc14 release is dependent upon Cdc5 in nocodazole-arrested cells; when *CDC5* is deleted, overexpressed Esp1 can no longer induce Cdc14 release. Cells are presimulated in metaphase arrest by nocodazole (*N* = 1) with no synthesis of Cdc5 (*k*_s,polo_ = 0) and no initial Cdc5 proteins. Then at *t* = 0 the rate of synthesis of Esp1 is increased 60-fold (*k*_s,esp_ = 0.078). (**C**) When *CLB5* is deleted, overexpressed Esp1 can induce ME. Reduction in Cdk activity by Clb5 deprivation allows for ME by increasing the phosphatase-to-kinase ratio, leading to activation of Cdh1. Simulation was done as in panel A, except that the synthesis rate of Clb2 was set to 80% of its basal value (*k*_s,b2_ = 0.024). (**D**) When Esp1 is inactive, Cdc14 cannot be released and the cell cannot exit from mitosis. It is assumed that separase is absent in *esp1-2^td^* mutant cells (*k_s,esp_* = 0). During the 120 min pre-simulation of Cdc20 block in metaphase, the rate of degradation of Esp1 was increased 10-fold, and the activity of Esp1 was lowered 10-fold. (*effesp* = 0.1, *k_d,esp_* = 0.028,). At *t* = 0, Cdc20 synthesis was induced, as usual.

Overexpressed separase is sufficient to trigger Cdc14 release (simulated in Figure 9A) in cells arrested in metaphase by depletion of Cdc20 [17], [56], [57]. These authors used the attenuated *GALS* promoter to overexpress Esp1, which allows viability and ME at 30°C. Cdc14 release in overexpressed Esp1 depends on Cdc5 activity (simulated in Figure 9B). *GALS-ESP1* cells do not exit from mitosis at 23°C as judged by cytokinesis and entry into the next cell cycle. Clb2 remains high because Cdc20 is inactive, and the Cdc14/Clb2 ratio may stay lower than the threshold to activate Cdh1. We are not exactly sure how temperature changes the phenotype. Temperature may alter the specific activity of Clb2/Cdk1 towards Cdh1, resulting in partially active Cdh1 at 23°C, which is unable to counteract all mitotic Cdk activity. In fact, when Cdk activity is reduced by deleting *CLB5* (simulated in Figure 9C) in *GAL-ESP1* cells, ME occurs in the absence of active Cdc20, as judged by cytokinesis and subsequent cell cycle [56]. In the simulation of *GAL-ESP1* at 23°C, we reduce by one-half the degradation rate of Clb2, resulting in higher Clb2, which prevents cells from exiting mitosis.

Esp1 is a protease that triggers chromosome segregation at anaphase onset by cleaving cohesin rings. Moreover, Esp1 downregulates PP2A by its nonproteolytic activity, allowing Cdc5 and Cdk kinases to phosphorylate Net1 and induce Cdc14 release. Our model incorporates both proteolytic and nonproteolytic functions of Esp1; nonproteolytic function of Esp1 leads to FEAR activation [17], [56], and its proteolytic activity is necessary for spindle elongation and MEN activation [57]. When Esp1 is inactive as in *esp1-2^td^* mutant (simulated in Figure 9D), there is no Cdc14 release, no cohesin cleavage, and no spindle elongation, resulting in inactive FEAR and MEN. Thus, cells fails to undergo cytokinesis, ME is blocked and cells remain arrested in mitosis [17], [58], [59].

**Figure 10:**
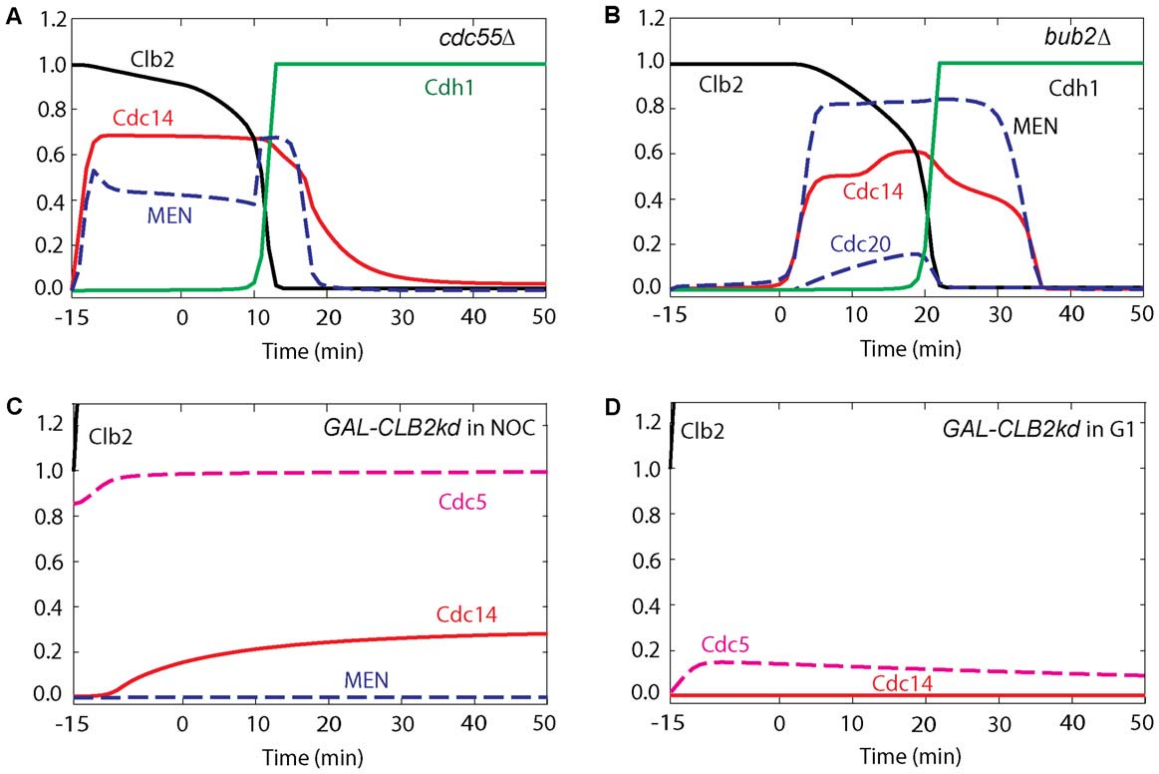
Mitotic progression of cells containing *CDC55*, *CLB2* and *BUB2* mutations. (**A**) In the *CDC55* deletion strain, Cdc14 is re-sequestered with a delay. Cdc20 block-and-release was simulated as usual, with [PP2A]_total_ = 0. (**B**) Cdc14 is released prematurely in *bub2Δ* cells. Cdc20 block-and-release was pre-simulated with the rates of Tem1 inactivation by Cdc14 and PP2A set to zero (*k*_i,tem′_ = *k*_i,tem″_ = 0). (**C**) In a nocodazole-arrested cell, overexpression of Clb2 induces Cdc14 release, and the cell arrests in telophase. Pre-simulation was done by setting *N* = 1 for 15 min. At *t* = 0, the rate of synthesis of Clb2 was increased 20-fold and the rates of degradation of Clb2 were set to zero (*N* = 1, *k*_d,b2′_ = *k*_d,b2″_ = 0, *k*_s,b2_ = 0.6). (**D**) Cdc14 is not released when Clb2 is overexpressed in G1 cells with Cdc5 inactive. This simulation was started from G1 initial conditions (low levels of Clb2, Cdc5 and Cdc14). At *t* = 0, the initial condition of Polo is set to 0.01, the synthesis rate of Clb2 is set to a large value and its degradation rate is set to zero (*k*_d,b2′_ = *k*_d,b2″_ = *k*_s,polo_ = 0, *k*_s,b2_ = 0.6).

Figure 10A shows that Cdc14 is prematurely released in *cdc55Δ* cells arrested in metaphase by depletion of Cdc20 [17], [35], [36]. The Cdc14 release in metaphase occurs due to high kinase activities (Cdc5 and Cdk/Clb2 phosphorylation of Net1) not counteracted by PP2A phosphatase activity. As we discuss in the following section, PP2A/Cdc55 promotes resequestration of Cdc14; thus, Cdc14's return to the nucleolus after ME is delayed in *cdc55Δ* cells [17]. In *bub2Δ* cells arrested in metaphase by depletion of Cdc20 (simulated in Figure 10B), Cdc14 is released prematurely mainly by MEN and returned with a delay [17], [20]. Overexpression of Clb2, which cannot be degraded by Cdc20 or Cdh1, promotes Net1 phosphorylation in cells arrested in metaphase (Figure 10C) but not in G1 cells (Figure 10D).

### Return of Cdc14 to the nucleolus depends on Cdc5 degradation and phosphatase activity

**Figure 11:**
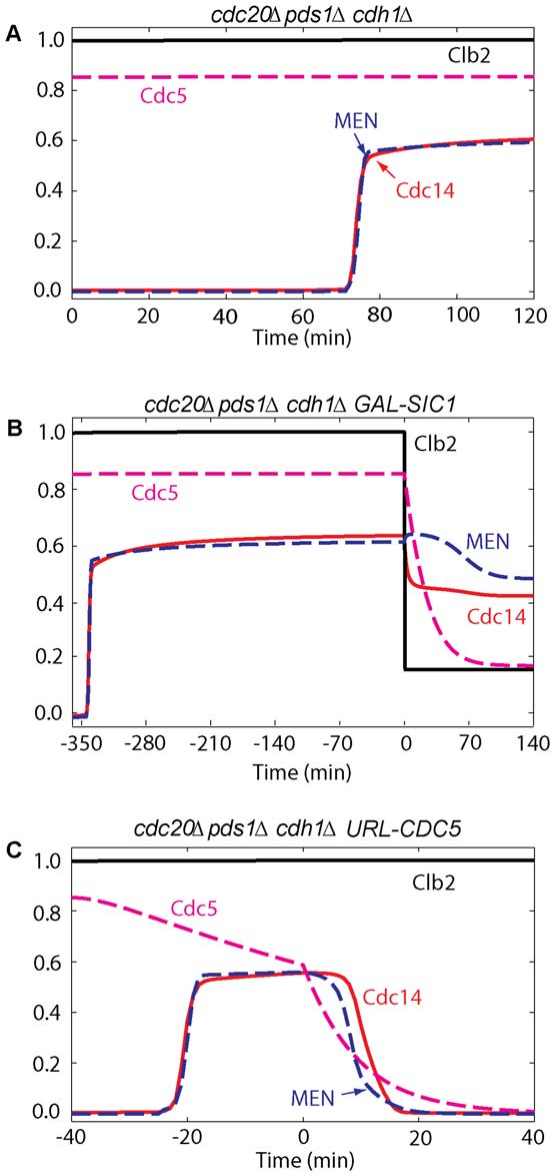
Inactive Clb2 is not required, whereas Polo inactivation is sufficient for Cdc14 re-sequestration to the nucleolus. (**A**) Cells of the triple-deletion strain *cdc20Δ pds1Δ cdh1Δ* arrest in telophase with Cdc14 released from the nucleolus. Simulation was done by setting to zero the rate of synthesis of Pds1, the total concentration of Cdh1, and the initial conditions of Cdh1, Pds1 and PE complex (*k*_s,pds_ = *CDH1T* = 0). (**B**) After 6 hours of telophase arrest, *cdc20Δ pds1Δ cdh1Δ* cells are subjected to Sic1 overexpression, and Cdc14 does not completely return to the nucleolus. Simulation was done as in panel A; after 6 hours *INH* was set to 5 to implement Cdk inhibition by Sic1. (**C**) In *cdc20Δ pds1Δ cdh1Δ* cells arrested in telophase, deprivation of Cdc5 causes return of Cdc14 to the nucleolus. Simulation was done as in panel A; after 40 min the rate of synthesis of Cdc5 was set to zero and the basal degradation rate of Cdc5 was increased 10-fold (*k*_s,polo_ = 0, *k*_d,polo_ = 0.1).

What is the mechanism promoting Cdc14 re-sequestration into the nucleolus after ME? At the onset of anaphase, kinase activities overcome phosphatase (PP2A) activity, and Cdc14 is released from both PRENT and PRENTP. As long as kinase activities remain high and PP2A activity is low (simulated in Figure 11A), Cdc14 remains released from the nucleolus [20]. So, either inactivation of kinases or elevated levels of PP2A after ME may contribute to Cdc14 return to the nucleolus. First, we investigated the role of degradation of Clb2 by Cdh1 in the return of Cdc14. Our simulations in Figure 11B, in agreement with the experiments [20], show that inhibition of Clb2-kinase activity (by its stoichiometric inhibitor, Sic1) is not enough to return Cdc14 to the nucleolus. Second, when we inhibit Cdk activity in telophase-arrested *MET-CDC20 pds1Δ cdh1Δ* cells, Cdc14 does not return to the nucleolus as reported in [20]. Therefore, inactivation of Cdk/Clb does not play a role in the return of Cdc14. Third, we explored the effect of removing Cdc5 from telophase-arrested *MET-CDC20 pds1Δ cdh1Δ* cells (simulated in Figure 11C), and found that Cdc14 returns to the nucleolus, in agreement with experiments [20]. Hence, Cdc5 is needed to sustain Cdc14 release during anaphase and telophase.

**Figure 12:**
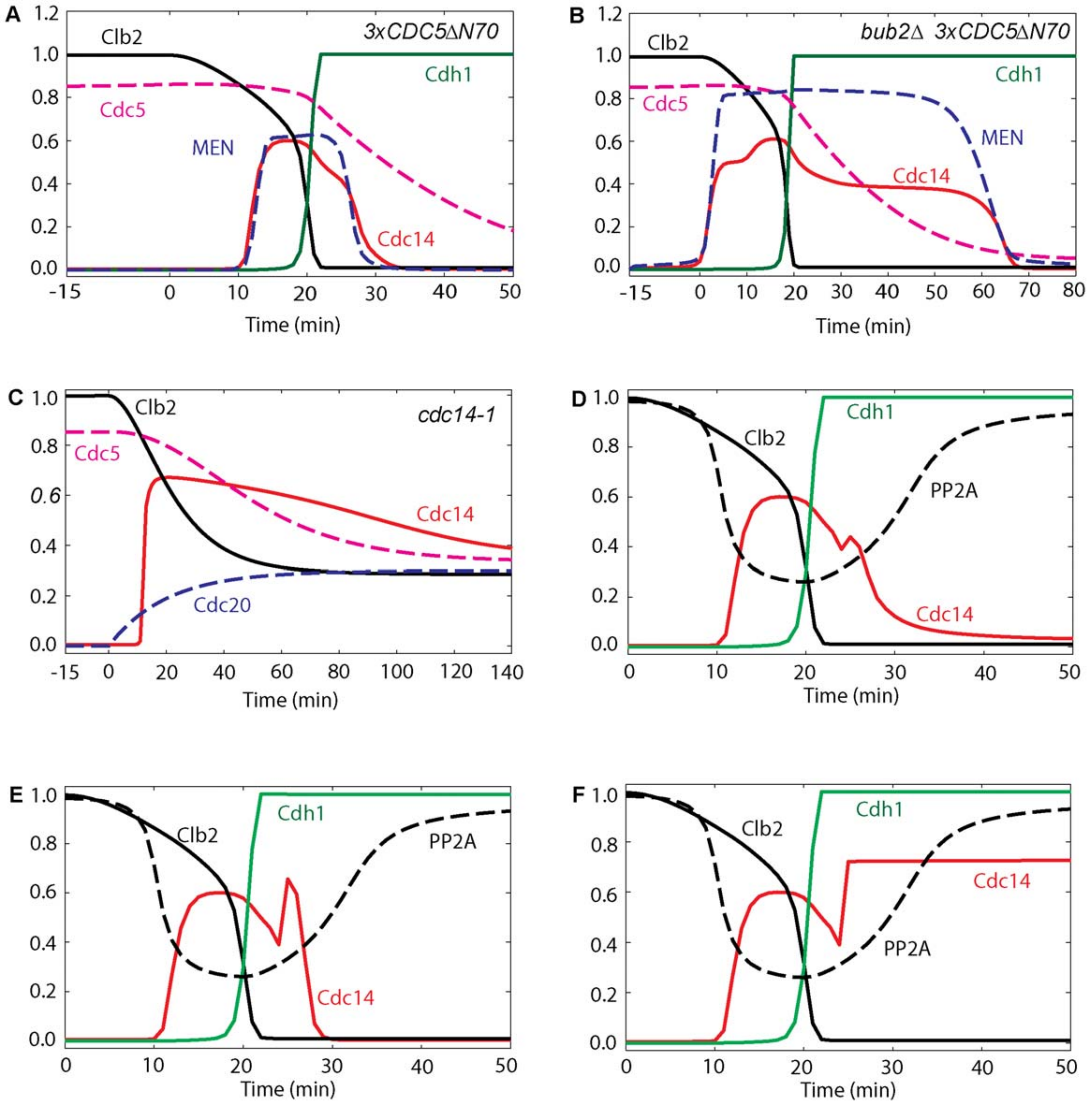
Effects of Cdc5 kinase and of Cdc14 and PP2A phosphatase activities on Cdc14 re-sequestration. A stabilized version of Cdc5 (3×*CDC5ΔN70*) causes a delay in Cdc14 re-sequestration both in wild-type cells (panel A) and *bub2Δ* background (panel B). (**A**) Cdc20 block-and-release was pre-simulated for 180 min with no degradation of Cdc5 (*k*_d,polo′_ = 0, *k*_s,polo_ = 0.011). (**B**) Simulation was done as in A with the rates of inactivation of Tem1 set to zero (*k*_i,tem′_ = *k*_i,tem″_ = 0). (**C**) Cdc14 stays released from the nucleolus in *cdc14-1* cells arrested in telophase. Cdc20 block-and-release was presimulated with no Cdc14 activity (*effc14* = 0). (**D**, **E** and **F**) Cdc20 block-and-release in wild-type cells; after 24 min (when cells start to enter G1 phase), either PP2A activity (panel D, *effpa* = 0) or Cdc14 activity (panel E, *effc14* = 0) was inhibited. In either case, Cdc14 is re-sequestered into the nucleoulus. In panel F, when both PP2A and Cdc14 phosphatase activities are inhibited after 24 min (*effpa* = *effc14* = 0), Cdc14 does not return to the nucleolus.

Simulations in Figure 12A–B show that the return of Cdc14 is delayed in *3×CDC5ΔN70* and in *bub2Δ 3×CDC5ΔN70* mutants when a proteolysis–resistant version of Cdc5 is expressed (*CDC5ΔN70* lacks destruction boxes) [20], confirming the importance of Cdc5 degradation for Cdc14 return. The degradation of Polo at ME not only reduces Net1 phosphorylation (by lowering the fluxes going into PRENT and PRENTP), but also inactivates Tem1 which in turn inactivates MEN. These effects are sufficient for returning Cdc14 to the nucleolus in wild-type cells.

Finally, in Figure 12C–F we investigate the roles of Cdc14 and PP2A phosphatase activities on Cdc14 re-sequestration after ME. Cells lacking Cdc14 phosphatase activity (c*dc14-1*) are arrested in telophase with elevated release of Cdc14 protein, low PP2A activity, and hyperphosphorylated Net1, in agreement with [55]. When we inhibit both PP2A and Cdc14 right after ME (simulated in Figure 12F), the model predicts that Cdc14 stays out, Cdh1 is on, and MEN turns off. When either Cdc14 or PP2A activity is inhibited after ME, the model predicts return of Cdc14 to the nucleolus with a delay (simulated in Figure 12D–E). Therefore, although either Cdc14 or PP2A is sufficient for re-sequestration of Cdc14 into RENT complexes, both Cdc14 and PP2A are needed for the timely return of Cdc14 to the nucleolus in wild-type cells. When Net1 dephosphorylation is completely blocked, Cdc14 does not return to the nucleolus.

### Cdc5 may phosphorylate Net1 *in vivo* to promote Cdc14 release

Cdc5 plays multiple roles during the cell cycle. As a component of MEN, Cdc5 promotes ME by phosphorylating (inhibiting) Bfa1, a negative regulator of MEN [39]. Since Cdc14 is transiently released in all MEN mutants except *cdc5*, Cdc5 is also a component of FEAR [44], [52]. In this section we present evidence that Cdc5's role in FEAR is to phosphorylate Net1.

Net1 is extensively phosphorylated by Cdc5 *in vitro* [23], and Cdc5 seems to influence the phosphorylation state of Net1 *in vivo* [23]. Cdc5 co-exists with Net1 in the nucleolus and may reduce the affinity between Net1 and Cdc14 [22]. In a *cdc5* mutant *(msd2-1)*, Net1 is not phosphorylated and Cdc14 is not released from the nucleolus during anaphase. Net1 is highly phosphorylated in nocodazole-arrested cells that are overexpressing Cdc5-dbΔ (a stable mutant form of Cdc5) [22], suggesting that Cdc5 phosphorylates Net1 *in vivo*.

When cells are arrested in metaphase by nocodazole [56], Cdc5 triggers Cdc14 release independently of Slk19, Esp1, Spo12, and other FEAR components, suggesting that Cdc5 has additional roles in ME beyond its phosphorylation of Bfa1. This result is confirmed by [55]. The additional role of Cdc5 to promote Cdc14 release may be the direct phosphorylation of Net1 because other known roles of Cdc5 are irrelevant. Cdc5's role in activating Tem1 and MEN is irrelevant under these conditions, because [23] showed that overexpression of Tem1, Cdc15, and Dbf2/Mob1 does not cause Cdc14 release in nocodazole-arrested cells. In fact, *CDC5* is the only gene whose overexpression causes premature release of Cdc14 during metaphase [23]. Overexpression of a proteolysis-resistant protein (*GAL-CDC5-dbΔ*) promotes Cdc14 release from the nucleolus in cells with short spindles after release from 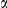-factor (Clb2 is low) or in cells arrested in metaphase by nocodazole (Clb2 is high) [23]. Therefore, overexpressed Cdc5 may induce Cdc14 release by phosphorylating Net1 independent of Clb2-kinase activity and independent of Cdc5's role in MEN or in cohesin cleavage and spindle elongation.

Overexpression of a stabilized form of Clb2, which cannot be degraded by Cdc20 or Cdh1, promotes Net1 phosphorylation and dispersal of Cdc14 in cells arrested in metaphase (simulated in Figure 10C) but not in G1 cells (simulated in Figure 10D) (compare Figure 4 in [55]). Because Cdc5 and MEN are inactive in G1 cells, this is further evidence that Clb2-dependent phosphorylation of Net1 alone is insufficient to convert Net1 into the inactive, phosphorylated form that releases Cdc14. The inactive form of Net1 may require phosphorylation by Cdc5 or MEN. According to our model (Figure 1), in the absence of Cdc5 activity, high Clb2 may phosphorylate RENT (converting it to RENTP), but Cdc14 is not released from this complex, as evidenced by these experiments.

**Figure 13:**
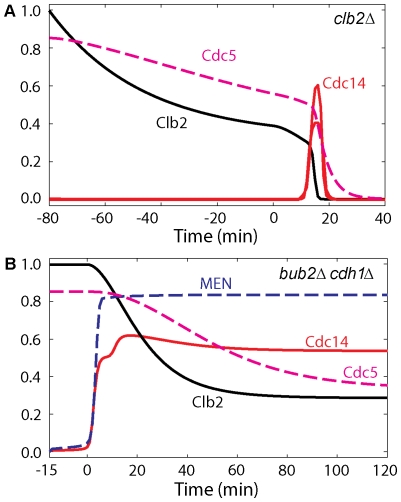
*clb2Δ* and *bub2Δ cdh1Δ* mutants. (**A**) When Clb2 is inhibited Cdc14 is released with a delay. Simulation was started at metaphase Cdc20 block for 80 min with rate of synthesis of Clb2 in the model decreased to 1/3 of baseline due to residual Clb1 activity (*k*_s,b2_ = 0.1) and Cdc20 was added back at time zero. (**B**) In *bub2Δ cdh1Δ* cells, Cdc14 stays released after ME. Simulation was done as wild-type cells except that rates of inactivation of Tem1 and total concentration of Cdh1 were set to zero (*k*_i,tem′_ = *k*_i,tem″_ = *CDH1T* = 0).

On the other hand, in the absence of Clb2-dependent phosphorylation of Net1, as in the *NET1-6cdk* mutant strain (simulated in Figure 5C) or in *clb2Δ* (simulated in Figure 13A), cells are still able to activate Cdc14 and exit from mitosis. As depicted in Figure 1, Cdc5 can still phosphorylate and inactivate RENT in the absence of Cdk/Clb2 phosphorylation of Net1.

Moreover, hyperphosphorylation of Net1 in *cdc14-1* cells is significantly reduced upon inactivation of Cdc5 [23], [55]. This hyperphosphorylation is not a consequence of MEN activity, since Cdc15 activity is low in *cdc14-1* cells. Hyperphosphorylation of Net1 could be a consequence of Cdc5 and/or Cdk activity. Since Net1 is not phosphorylated in the *cdc5-1 cdc14-1* double mutant (simulated in Figure 4F) [55], Cdc14 release in *cdc14-1* may be attributable solely to Net1 phosphorylation by Cdc5.

Overexpression of Esp1 induces Cdc14 release in cells arrested in metaphase by nocodazole [17], [20], [30], [56]. When Cdc5 is inhibited (*GAL-ESP1 cdc5as-1*), Cdc14 is no longer released (simulated in Figure 9B) [20]. Although PP2A activity is very low (due to high levels of Esp1), Net1 phosphorylation by endogenous Cdk/Clb2 alone is not sufficient to induce Cdc14 release.

Cells of the *cdc20Δ pds1Δ cdh1Δ* mutant strain (simulated in Figure 11A) are arrested in telophase with high Clb2 activity, low PP2A activity, and elevated release of Cdc14 (see Figure 9 in [20]. After Cdc5 activity is eliminated, Cdc14 returns to the nucleolus, suggesting that high Clb2 activity alone cannot inactivate Net1 in the absence of Cdc5 activity.

Considering Net1 phosphorylation in FEAR as dependent only on Cdk activity would be inadequate to explain the mutant phenotypes presented above. The facts that Cdc5 is both necessary and sufficient for Cdc14 release [20], that Cdc5 influences phosphorylation state of Net1 *in vivo* [23], that Net1 is extensively phosphorylated by Cdc5 *in vitro* [23], and the evidences given in this section suggest that Cdc5 may promote Cdc14 release by direct phosphorylation of Net1, as proposed in Figure 1.

### The model predicts phenotypes of novel mutants

In each simulation of an experimentally characterized mutant genotype, we have compared some of our simulation results against relevant observations. Other details of the simulations may be considered as predictions, because the experimental studies did not report the relevant information. These predictions are useful for future experimental studies of mutant strains that have already been characterized. In addition, our model can be used to predict the phenotypes of mutant strains that have not yet been described in the literature (see Table 1). Our predictions for these novel mutants are described in detail on our website http://mpf.biol.vt.edu/research/mitotic_exit_model/pp/index.php.

**Table 1:** Prediction of mutant phenotypes.

Genotype	Phenotype
*GAL-CDC5*	Cdc14 starts to be released in metaphase, ME occurs earlier
*GAL-CDC5 cdc15-as1 GAL-PDS1*	Cdc14 is released transiently and returns back, no ME
*GAL-CDC5 cdc15-2 NET1-6cdk*	Cdc14 is released and cells exit with a delay
*GAL-CDC5 GAL-PDS1*	Cdc14 is released transiently and returns back, no ME
*wild-type then cdc14-3 pp2a*	Cdc14 stays out after ME although MEN turns off
*wild-type then cdc14-3*	Cdc14 returns back after ME
*wild-type then pp2a*	Cdc14 returns back after ME
*cdc15-2 cdc14-3*	Cdc14 is not sequestered back into the nucleolus in telophase arrest
*cdc15-2 pp2a*	ME occurs in *cdc15-2* cells after PP2A is inactivated at 20 min
*cdc15-2 pp2a NET1-6cdk*	Cdc14 is not sequestered back into the nucleolus in telophase arrest
*bub2Δ cdh1Δ GAL-SIC1*	Cdc14 re-sequestered back after Cdk inactivation by Sic1
*bub2Δ cdc5-1*	no Cdc14 in metaphase, delay in Cdc14 release, ME with a delay
*bub2Δ cdc5-as1*	no Cdc14 release, no ME
*bub2Δ NET1-6cdk*	similar to *bub2-del mutant*
*GAL-ESP1 URL-CDC5*	Cdc14 sequestered back after Cdc5 inactivation, no ME
*GAL-ESP1 clb2Δ*	GAL-ESP1 cells exit from mitosis after Clb2 degradation
*GAL-ESP1 cdc15-2 clb2Δ*	no ME when MEN is inactive, and Cdc14 stays out
*GALS-ESP1 cdc14-3 cdc20Δ*	Cdc14 is released, stays out, no ME
*GALS-ESP1 NET1-6cdk cdc20Δ*	Cdc14 is released, stays out, no ME
*GAL-PDS1-mdb cdc55-del*	ME occurs similar to *cdc55-del*
*CLB2-dbΔ cdc5-as1*	Cdc14 is not released, no ME

Simulations of some of these mutants are provided on the model webpage.

## Discussion

Molecular cell biologists have collected a large amount of data about the proteins, genes and biochemical reactions involved in the regulation of mitotic exit (ME) in *S. cerevisiae*. Using nonlinear differential equations, we have developed a realistic, quantitative model of the molecular control system governing ME in yeast based on this published data. Our model provides an opportunity to analyze the system-level dynamics of ME events, to investigate *in silico* our hypotheses about the molecular machinery of ME, and to suggest new experiments that test predictions of the model. The proposed model should be viewed as an evolving hypothesis to be continually revised and improved based on new observations about the molecular control of ME in budding yeast.

In particular, our model deals with the release of Cdc14 phosphatase from the nucleolus during anaphase and telophase, as a result of the kinase activities of Cdk/Clb2 and Polo, the activation of Esp1 and inhibition of PP2A, sister chromatid separation and spindle elongation, and the interconnectivity of the FEAR and MEN pathways. We explored the roles of Cdc5 (Polo kinase) and Esp1 in FEAR and MEN. We propose that Cdc5 phosphorylates Net1 *in vivo*, inducing Cdc14 release both in early and late anaphase. We propose that Cdc5 also activates Tem1 (a G-protein in the MEN pathway) in two ways: by phosphorylating and inactivating Bub2-Bfa1 (Tem1's GAP), and by promoting spindle elongation, which brings Lte1 (Tem1's GEF) into contact with Tem1. Degradation or inactivation of Cdc5 is sufficient to silence both FEAR and MEN. Therefore, once Cdc5 activity drops, Cdc14 returns to the nucleolus. Both Cdc14 and PP2A phosphatases promote the re-sequestration of Cdc14 into the nucleolus.

In addition, we integrate both proteolytic and nonproteolytic functions of Esp1 into the kinetics of ME. FEAR activation requires the nonproteolytic function (inactivation of PP2A), whereas MEN activation requires its proteolytic function (cohesin cleavage, spindle elongation and subsequent activation of Tem1). Overexpressed Esp1 induces Cdc14 release and ME in the absence of Cdc20, but it requires Cdc5, an intact spindle, and MEN activation.

Recently Vinod *et al.* [19] published a model of ME in budding yeast focusing on the catalytic and non-catalytic roles of separase (Esp1) and on Cdc14 endocycles [24], [25]. Both Vinod's model and our model are based on Queralt *et al.* [17], but they address somewhat different aspects of ME in budding yeast. Our model addresses a broad range of ME experiments (the model webpage presents more than 100 mutant simulations). Vinod's model accounts for Cdc14 oscillations observed in the presence of non-degradable Clb2 [24], [25], but we have been unable to simulate Cdc14 oscillations under these conditions without compromising our correct simulations of other important ME mutant phenotypes. In our opinion, further experimental and modeling studies are needed to better understand Cdc14 endocycles, to integrate them into a comprehensive model of the majority of ME mutants, and to investigate their relevance for yeast cell-cycle control.

Another recent paper [60] presents a model of the anaphase switch (the metaphase-anaphase transition and its associated checkpoint) but does not address other details of exit from mitosis. A reasonable goal for future modeling work will be to incorporate this model of the anaphase switch into our model of mitotic exit (borrowing good ideas from Vinod's model), and then incorporating the entire ME story into Chen's 2004 model of the full cell cycle of budding yeast. At the same time, it will be useful to add a module describing the morphogenetic checkpoint in the budding yeast cell cycle (e.g., Ciliberto *et al.* [61]) and an improved model of Whi5-SBF interactions at the Start transition (e.g., Barberis *et al.* [62]).

Our model of ME in budding yeast organizes a large body of experimental information in a comprehensive and comprehensible manner. We believe it provides an accurate and predictive mathematical description of molecular events regulating ME events in budding yeast. We hope that this model, together with other quantitative models of yeast cell cycle controls, will provide a solid basis to develop models of cell cycle progression in the cells of higher eukaryotes, including humans.

## Methods

Based on a thorough review of the experimental literature and an earlier model of ME in budding yeast [17], we propose an extended ‘wiring diagram’ for the molecular regulation of FEAR and MEN pathways in *S. cerevisiae* (Figure 1). In the context of certain standard modeling assumptions (Text S2), we translate the wiring diagram into a set of nonlinear ordinary differential equations (Table S1) describing the production, degradation, activation, inhibition, binding, release, phosphorylation, dephosphorylation, localization, and delocalization of ME proteins and physiological variables. The ME proteins tracked by our model are: Clb2, Cdc5, Cdc14, Cdh1, Cdc20, Esp1, Cdc55/PP2A, Pds1, Net1, Tem1, and Cdc15. The physiological variables are: *C* (cohesin cleavage by separase), and *S* (spindle elongation driving sister chromatid separation, after cohesin cleavage). Simulation of the 18 ODEs in Table S1 requires numerical settings for 59 kinetic constants (*k's*) and 8 binding constants (*J's*), and specification of appropriate initial conditions of the variables. Parameter values (Table S2) were chosen to provide a good fit of the model to available experimental observations of wild-type and mutant cells. We do not assert that this set of parameter values is optimal in any sense. Initial conditions (Table S3) were chosen to represent steady state values of model variables in metaphase of wild-type cells.

Given these settings, the ODEs were solved numerically with two software packages: XPPAut and PET, freely available at http://www.math.pitt.edu/~bard/xpp/xpp.html and http://mpf.biol.vt.edu/pet/. For further explanation and justification of our modeling methods, see [9].

To present our model in a complete and systematic form, we have developed a website (http://mpf.biol.vt.edu/research/mitotic_exit_model/pp/) that includes a full description of the model, simulations of wild-type cells, model files and all relevant mutants. The web site is intended to help molecular biologists to design new experiments and mathematical modelers to explore the model in greater detail. Text S3 provides a machine-readable file for reproducing our simulations in other modeling environments.

The phenotypes of relevant mutants were collected from the literature. To simulate each mutant, we use exactly the same equations (Table S1) and ‘basal’ parameter values (Table S2) except for those parameters directly affected by the mutation (see model webpage).

The standard experimental protocol for studying ME events in budding yeast is ‘Cdc20 block-and-release’, using the strain *cdc20Δ GAL-CDC20* (which is ‘wild-type’ for the purposes of this paper). Cells grown in glucose medium arrest in metaphase because they are depleted of Cdc20. At *t* = 0, cells are transferred to galactose medium. The newly synthesized Cdc20 protein is ‘active’ because the replicated chromosomes have been properly aligned on the mitotic spindle in glucose medium. Additional mutations are added on top of the Cdc20 block-and-release strain in the model exactly as in the experiments. For each of these mutant strains, we pre-simulate the Cdc20-deletion cells, with the additional mutations, for 15 min (or as prescribed in the experimental conditions) and then add back Cdc20 (setting the synthesis rate of Cdc20 to the wild-type value, 0.015). For a gene deletion, the rate of synthesis of the corresponding protein is set to zero. For gene overexpression, an additional constant rate of synthesis of the corresponding protein is introduced into the equations, because proteins are typically overexpressed from an extra copy of the gene under control of an inducible promoter. For temperature-sensitive mutants, the relevant rate constant(s) retains its wild-type value at the permissive temperature and is set to zero (or to 10% of its basal value) at the restrictive temperature. For partial deletions, the relevant parameter value is assumed to lie between 0 and 100% of the wild-type (basal) value, according to the experimental characterization of the mutation.

## Supporting Information

Table S1 **Differential equations of the model.**
(PDF)

Table S2 **Basal parameter values for wild-type cells.**
(PDF)

Table S3 **Initial values of model variables.**
(PDF)

Text S1 **A consensus picture of mitotic exit in budding yeast.**
(PDF)

Text S2 **Assumptions of the Model.**
(PDF)

Text S3 **MitoticExit.ode.**
(ODE)
