# Supplementary material for: A Mathematical Model of Mitotic Exit in Budding Yeast: The Role of Polo Kinase
Source: PLoS One. 2012 Feb 23;7(2):e30810. doi: 10.1371/journal.pone.0030810 (PMC3285609; doi:10.1371/journal.pone.0030810)
Supplement: Table S1 — Differential equations of the model. (PDF) [file pone.0030810.s001.pdf]

Table S1: Equations of the Model

$$\frac{d[\text{Clb2}]}{dt} = k_{\text{s,b2}} - V_{\text{d,b2}} \cdot [\text{Clb2}]$$

$$\frac{d[\text{Cdc20}]}{dt} = k_{\text{s,20}} - V_{\text{d,20}} \cdot [\text{Cdc20}]$$

$$\frac{d[\text{Cdh1}]}{dt} = \frac{V_{\text{a,cdh}} \cdot \left([\text{Cdh1}]_T - [\text{Cdh1}]\right)}{J_{\text{a,cdh}} + \left([\text{Cdh1}]_T - [\text{Cdh1}]\right)} - \frac{V_{\text{i,cdh}} \cdot [\text{Cdh1}]}{J_{\text{i,cdh}} + [\text{Cdh1}]}$$

$$\frac{d[\text{Pds1}]}{dt} = k_{\text{s,pds}} - V_{\text{d,pds}} \cdot [\text{Pds1}] - l_{\text{as,pds}} \cdot [\text{Pds1}] \cdot [\text{Esp1}] + l_{\text{d,pds}} \cdot [\text{PE}] + k_{\text{d,esp}} \cdot [\text{PE}]$$

$$\frac{d[\text{Esp1}]}{dt} = -l_{\text{as,pds}} \cdot [\text{Pds1}] \cdot [\text{Esp1}] + l_{\text{d,pds}} \cdot [\text{PE}] + k_{\text{s,esp}} - k_{\text{d,esp}} \cdot [\text{Esp1}] + V_{\text{d,pds}} \cdot [\text{PE}]$$

$$\frac{d[\text{PE}]}{dt} = l_{\text{as,pds}} \cdot [\text{Pds1}] \cdot [\text{Esp1}] - l_{\text{d,pds}} \cdot [\text{PE}] - V_{\text{d,pds}} \cdot [\text{PE}] - k_{\text{d,esp}} \cdot [\text{PE}]$$

$$\frac{d[\text{Poloi}]}{dt} = k_{\text{s,polo}} - V_{\text{d,polo}} \cdot [\text{Poloi}] - \frac{V_{\text{a,polo}} \cdot [\text{Poloi}]}{J_{\text{a,polo}} + [\text{Poloi}]} + \frac{k_{\text{i,polo}} \cdot [\text{Polo}]}{J_{\text{i,polo}} + [\text{Polo}]}$$

$$\frac{d[\text{Polo}]}{dt} = \frac{V_{\text{a,polo}} \cdot [\text{Poloi}]}{J_{\text{a,polo}} + [\text{Poloi}]} - \frac{k_{\text{i,polo}} \cdot [\text{Polo}]}{J_{\text{i,polo}} + [\text{Polo}]} - V_{\text{d,polo}} \cdot [\text{Polo}]$$

$$\frac{d[\text{Tem1a}]}{dt} = -l_{\text{a,men}} \cdot [\text{Tem1a}] \cdot [\text{Cdc15a}] + l_{\text{d,men}} \cdot [\text{MEN}] + \frac{V_{\text{a,tem}} \cdot [\text{Tem1i}]}{J_{\text{tem}} + [\text{Tem1i}]} - \frac{V_{\text{i,tem}} \cdot [\text{Tem1a}]}{J_{\text{tem}} + [\text{Tem1a}] + [\text{MEN}]} + \frac{V_{\text{i,c15}} \cdot [\text{MEN}]}{J_{\text{c15}} + [\text{Cdc15a}] + [\text{MEN}]}$$

$$\frac{d[\text{Cdc15a}]}{dt} = -l_{\text{a,men}} \cdot [\text{Tem1a}] \cdot [\text{Cdc15a}] + l_{\text{d,men}} \cdot [\text{MEN}] + \frac{V_{\text{i,tem}} \cdot [\text{MEN}]}{J_{\text{tem}} + [\text{Tem1a}] + [\text{MEN}]} + \frac{V_{\text{a,c15}} \cdot [\text{Cdc15i}]}{J_{\text{c,15}} + [\text{Cdc15i}]} - \frac{V_{\text{i,c15}} \cdot [\text{Cdc15a}]}{J_{\text{c,15}} + [Cdc15a] + [\text{MEN}]}$$

$$\frac{d[\text{MEN}]}{dt} = l_{\text{a,men}} \cdot [\text{Tem1a}] \cdot [\text{Cdc15a}] - l_{\text{d,men}} \cdot [\text{MEN}] - \frac{V_{\text{i,tem}} \cdot [\text{MEN}]}{J_{\text{tem}} + [\text{Tem1a}] + [\text{MEN}]} - \frac{V_{\text{i,c15}} \cdot [\text{MEN}]}{J_{\text{c,15}} + [\text{Cdc15a}] + [\text{MEN}]}$$

$$\frac{d[\text{Cdc14}]}{dt} = -l_{\text{a,1}} \cdot [\text{Cdc14}] \cdot [\text{Net1}] + l_{\text{d,1}} \cdot [\text{RENT}] - l_{\text{a,2}} \cdot [\text{Cdc14}] \cdot [\text{Net1P}] + l_{\text{d,2}} \cdot [\text{RENTP}] - l_{\text{a,3}} \cdot [\text{Cdc14}] \cdot [\text{PNet1}] + l_{\text{d,3}} \cdot [\text{PRENT}]$$
$$- l_{\text{a,4}} \cdot [\text{Cdc14}] \cdot [\text{PNet1P}] + l_{\text{d,4}} \cdot [\text{PRENTP}]$$

$$\frac{d[\mathrm{Net1}]}{dt} = -l_{\mathrm{a,1}} \cdot [\mathrm{Cdc14}] \cdot [\mathrm{Net1}] + l_{\mathrm{d,1}} \cdot [\mathrm{RENT}] - \frac{V_{\mathrm{k,12}} \cdot [\mathrm{Net1}]}{J_{12} + [\mathrm{Net1}] + [\mathrm{RENT}]} + \frac{V_{\mathrm{p,21}} \cdot [\mathrm{Net1P}]}{J_{12} + [\mathrm{Net1P}] + [\mathrm{RENTP}]}$$
$$- \frac{V_{k,13} \cdot [Net1]}{J_{13} + [\mathrm{Net1}] + [\mathrm{RENT}]} + \frac{V_{\mathrm{p,31}} \cdot [\mathrm{PNet1}]}{J_{13} + [\mathrm{PNet1}] + [\mathrm{PRENT}]}$$

$$\frac{d[\mathrm{PNet1}]}{dt} = -l_{\mathrm{a,3}} \cdot [\mathrm{Cdc14}] \cdot [\mathrm{PNet1}] + l_{\mathrm{d,3}} \cdot [\mathrm{PRENT}] - \frac{V_{\mathrm{k,34}} \cdot [\mathrm{PNet1}]}{J_{34} + [\mathrm{PNet1}] + [\mathrm{PRENT}]} + \frac{V_{\mathrm{p,43}} \cdot [\mathrm{PNet1P}]}{J_{34} + [\mathrm{PNet1P}] + [\mathrm{PRENTP}]}$$
$$+ \frac{V_{\mathrm{k,13}} \cdot [\mathrm{Net1}]}{J_{13} + [\mathrm{Net1}] + [\mathrm{RENT}]} - \frac{V_{\mathrm{p,31}} \cdot [\mathrm{PNet1}]}{J_{13} + [\mathrm{PNet1}] + [\mathrm{PRENT}]}$$

$$\frac{d[\mathrm{PNet1P}]}{dt} = -l_{\mathrm{a,4}} \cdot [\mathrm{Cdc14}] \cdot [\mathrm{PNet1P}] + l_{\mathrm{d,4}} \cdot [\mathrm{PRENTP}] + \frac{V_{\mathrm{k,34}} \cdot [\mathrm{PNet1}]}{J_{34} + [\mathrm{PNet1}] + [\mathrm{PRENT}]}$$
$$- \frac{V_{\mathrm{p,43}} \cdot [\mathrm{PNet1P}]}{J_{34} + [\mathrm{PNet1P}] + [\mathrm{PRENTP}]} + \frac{V_{\mathrm{k,24}} \cdot [\mathrm{Net1P}]}{J_{24} + [Net1P] + [\mathrm{RENTP}]} - \frac{V_{\mathrm{p,42}} \cdot [\mathrm{PNet1P}]}{J_{24} + [\mathrm{PNet1P}] + [\mathrm{PRENTP}]}$$

$$\frac{d[\mathrm{RENT}]}{dt} = l_{\mathrm{a,1}} \cdot [\mathrm{Cdc14}] \cdot [\mathrm{Net1}] - l_{\mathrm{d,1}} \cdot [\mathrm{RENT}] - \frac{V_{\mathrm{k,12}} \cdot [\mathrm{RENT}]}{J_{12} + [\mathrm{Net1}] + [\mathrm{RENT}]}$$
$$+ \frac{V_{\mathrm{p,21}} \cdot [\mathrm{RENTP}]}{J_{12} + [\mathrm{Net1P}] + [\mathrm{RENTP}]} - \frac{V_{\mathrm{k,13}} \cdot [\mathrm{RENT}]}{J_{13} + [\mathrm{Net1}] + [\mathrm{RENT}]} + \frac{V_{\mathrm{p,31}} \cdot [\mathrm{PRENT}]}{J_{13} + [\mathrm{PNet1}] + [\mathrm{PRENT}]}$$

$$\frac{d[\mathrm{PRENT}]}{dt} = l_{\mathrm{a,3}} \cdot [\mathrm{Cdc14}] \cdot [\mathrm{PNet1}] - l_{\mathrm{d,3}} \cdot [\mathrm{PRENT}] - \frac{V_{\mathrm{k,34}} \cdot [\mathrm{PRENT}]}{J_{34} + [\mathrm{PNet1}] + [\mathrm{PRENT}]} + \frac{V_{\mathrm{p,43}} \cdot [\mathrm{PRENTP}]}{J_{34} + [\mathrm{PNet1P}] + [\mathrm{PRENTP}]}$$
$$+ \frac{V_{\mathrm{k,13}} \cdot [\mathrm{RENT}]}{J_{13} + [\mathrm{Net1}] + [\mathrm{RENT}]} - \frac{V_{\mathrm{p,31}} \cdot [\mathrm{PRENT}]}{J_{13} + [\mathrm{PNet1}] + [\mathrm{PRENT}]}$$

$$\frac{d[\mathrm{PRENTP}]}{dt} = l_{\mathrm{a,4}} \cdot [\mathrm{Cdc14}] \cdot [\mathrm{PNet1P}] - l_{\mathrm{d,4}} \cdot [\mathrm{PRENTP}] - \frac{V_{\mathrm{k,34}} \cdot [\mathrm{PRENT}]}{J_{34} + [\mathrm{PNet1}] + [\mathrm{PRENT}]} + \frac{V_{\mathrm{p,43}} \cdot [\mathrm{PRENTP}]}{J_{34} + [\mathrm{PNet1P}] + [\mathrm{PRENTP}]}$$
$$+ \frac{V_{\mathrm{k,24}} \cdot [\mathrm{RENTP}]}{J_{24} + [\mathrm{Net1P}] + [\mathrm{RENTP}]} - \frac{V_{\mathrm{p,42}} \cdot [\mathrm{PRENTP}]}{J_{24} + [\mathrm{PNet1P}] + [\mathrm{PRENTP}]}$$

$$[\mathrm{CDK}] = \frac{[\mathrm{Clb2}]}{1.0 + INH}$$

$$[\mathrm{PP2A}] = \frac{1.0 + kpp \cdot ki \cdot [\mathrm{ACTEsp1}]}{1.0 + ki \cdot [\mathrm{ACTEsp1}]} \cdot PP2AT$$

$$S = \exp((-ks) * (t - tm)) * (1.0 - N) * C * (1.0 - SCCRRDD)$$

$$V_{\mathrm{d,b2}} = k_{\mathrm{d,b2}} + k_{\mathrm{d,b2'}} \cdot [\mathrm{Cdc20}] + k_{\mathrm{d,b2''}} \cdot [\mathrm{Cdh1}]$$

$$V_{\mathrm{d,20}} = k_{\mathrm{d,20}} + k_{\mathrm{d,20'}} \cdot [\mathrm{Cdh1}]$$

$$V_{\mathrm{i,cdh}} = k_{\mathrm{k,cdh}} + k_{\mathrm{k,cdh'}} \cdot [\mathrm{CDK}]$$

$$V_{a,cdh} = k_{p,cdh} + k_{p,cdh'} \cdot [\text{ACTCdc14}]$$

$$V_{d,pds} = k_{d,pds} + k_{d,pds'} \cdot [\text{Cdc20}]$$

$$V_{a,polo} = k_{a,polo} + k_{a,polo'} \cdot [\text{CDK}]$$

$$V_{d,polo} = k_{d,polo} + k_{d,polo'} \cdot [\text{Cdh1}]$$

$$V_{a,tem} = k_{a,tem} + k_{a,tem'} \cdot [\text{ACTPOLO}] + k_{a,tem''} \cdot \text{S} \cdot [\text{ACTPOLO}]$$

$$V_{i,tem} = k_{i,tem} + k_{i,tem'} \cdot [\text{ACTPP2A}] + k_{i,tem''} \cdot [\text{ACTCdc14}]$$

$$V_{a,c15} = k_{a,c15} + k_{a,c15'} \cdot [\text{ACTCdc14}]$$

$$V_{i,c15} = k_{i,c15} + k_{i,c15'} \cdot [\text{CDK}]$$

$$V_{k,12} = k_{k,12} \cdot [\text{CDK}]$$

$$V_{p,21} = k_{p,21} \cdot [\text{ACTPP2A}] + k_{p,21'} \cdot [\text{ACTCdc14}]$$

$$V_{k,34} = k_{k,34} \cdot [\text{CDK}]$$

$$V_{p,43} = k_{p,43} \cdot [\text{ACTPP2A}] + k_{p,43'} \cdot [\text{ACTCdc14}]$$

$$V_{k,13} = k_{k,13} \cdot effc15 \cdot [\text{CDC15a}] + k_{k,13'} \cdot [\text{ACTPOLO}] + k_{k,13''} \cdot [\text{ACTMEN}]$$

$$V_{p,31} = k_{p,31} \cdot [\text{ACTPP2A}] + \text{k}_{p,31'} \cdot [\text{ACTCdc14}]$$

$$V_{k,24} = k_{k,24} \cdot [\text{ACTPOLO}] + k_{k,24'} \cdot [\text{ACTMEN}]$$

$$V_{p,42} = k_{p,42} \cdot [\text{ACTPP2A}] + k_{p,42'} \cdot [\text{ACTCdc14}]$$

$$[\text{PDS1T}] = [\text{PDS1}] + [\text{PE}]$$

$$[\text{Esp1T}] = [\text{Esp1}] + [\text{PE}]$$

$$[\text{ACTCdc14}] = effc14 \cdot [\text{Cdc14}]$$

$$\text{ACTMEN} = effc15 \cdot [\text{MEN}]$$

$$[\text{ACTPOLO}] = effpol \cdot [\text{POLO}]$$

$$[\text{ACTEsp1}] = effesp \cdot [\text{Esp1}]$$

$$[\text{ACTPP2A}] = \textit{effppa} \cdot [\text{PP2A}]$$
