# Supplementary material for: A Mathematical Model of Mitotic Exit in Budding Yeast: The Role of Polo Kinase
Source: PLoS One. 2012 Feb 23;7(2):e30810. doi: 10.1371/journal.pone.0030810 (PMC3285609; doi:10.1371/journal.pone.0030810)
Supplement: Table S2 — Basal parameter values for wild-type cells. (PDF) [file pone.0030810.s002.pdf]

Table S2: Basal parameter values for the wild-type cells

$k_{s,b2}$ = 0.03	J_{polo} = 0.25	$k_{p,43'}$ = 0.2
INH = 0	$k_{a,tem}$ = 0	J_{34} = 1
$k_{d,b2}$ = 0.03	$k_{a,tem'}$ = 2.8	$k_{k,13}$ = 0.4
$k_{d,b2'}$ = 0.25	$k_{a,tem''}$ = 13	$k_{k,13'}$ = 2.3
$k_{d,b2''}$ = 3	$k_{i,tem}$ = 0.35	$k_{k,13''}$ = 35
kpp = 0.1	$k_{i,tem'}$ = 4.5	$k_{p,31}$ = 2.6
ki = 20	$k_{i,tem''}$ = 2.8	$k_{p,31'}$ = 15
$PP2AT$ = 1	J_{tem} = 0.05	J_{13} = 0.1
$k_{s,20}$ = 0	$l_{a,men}$ = 15	$k_{k,24}$ = 5.5
$k_{d,20}$ = 0.05	$l_{d,men}$ = 0.1	$k_{k,24'}$ = 35
$k_{d,20'}$ = 1.5	$k_{a,c15}$ = 0	$k_{p,42}$ = 3.8
$J_{c,dh}$ = 0.001	$k_{a,c15'}$ = 1.5	$k_{p,42'}$ = 3
$k_{p,cdh}$ = 0.035	$k_{i,c15}$ = 0	J_{24} = 0.05
$k_{p,cdh'}$ = 1.2	$k_{i,c15'}$ = 0.09	$Cdh1T$ = 1
$k_{k,cdh}$ = 0	J_{c15} = 0.2	$Tem1T$ = 1
$k_{k,cdh'}$ = 1.22	$l_{a,1}$ = 300	$Cdc15T$ = 1
$k_{s,pds}$ = 0.03	$l_{d,1}$ = 1	$Cdc14T$ = 1
$k_{d,pds}$ = 0.03	$l_{a,2}$ = 10	$NET1T$ = 2

$k_{d,pds'}$ = 2	$l_{d,2}$ = 0.2	C = 0
$l_{a,pds}$ = 500	$l_{a,3}$ = 1	S = 0
$l_{d,pds}$ = 1	$l_{d,3}$ = 4	N = 0
$k_{s,esp}$ = 0.0013	$l_{a,4}$ = 1	k_{s} = 0
$k_{d,esp}$ = 0.004	$l_{d,4}$ = 1	t_{m} = 0
$k_{s,polo}$ = 0.01	$k_{k,12}$ = 1	*effc14* = 1
$k_{d,polo}$ = 0.01	$k_{p,21}$ = 40	effc15= 1
$k_{d,polo'}$ = 0.15	$k_{p,21'}$ = 5	*effpol* = 1
$k_{a,polo}$ = 0.0065	J_{12} = 2	*effppa* = 1
$k_{a,polo'}$ = 0.09	$k_{k,34}$ = 2	*effesp* = 1
$k_{i,polo}$ = 0.035	$k_{p,43}$ = 0.6	*SCCRRDD* = 0
