# Supplementary material for: A Mathematical Model of Mitotic Exit in Budding Yeast: The Role of Polo Kinase
Source: PLoS One. 2012 Feb 23;7(2):e30810. doi: 10.1371/journal.pone.0030810 (PMC3285609; doi:10.1371/journal.pone.0030810)
Supplement: Table S3 — Initial values of model variables. (PDF) [file pone.0030810.s003.pdf]

Table S3: Initial values of model variables

[Clb2] = 0.996	[Tem1a] = 0.044	[POLO] = 0.852	[Cdc14] = 0.005
[Cdc20] = 0	[Cdc15a] = 0.014	[PRENTP] = 0.02	[PRENT] = 0.062
[Cdh1] = 3.4E-5	[MEN] = 1.9E-4	[Pds1] = 0.675	[PNet1] = 0.099
[Esp1] = 9.9E-4	[Net1] = 0.829	[PE] = 0.324	[RENT] = 0.899
[POLOi] = 0.146	[PNet1P] = 0.051		
