# Supplementary material for: A Mathematical Model of Mitotic Exit in Budding Yeast: The Role of Polo Kinase
Source: PLoS One. 2012 Feb 23;7(2):e30810. doi: 10.1371/journal.pone.0030810 (PMC3285609; doi:10.1371/journal.pone.0030810)
Supplement: Text S1 — A consensus picture of mitotic exit in budding yeast. (PDF) [file pone.0030810.s004.pdf]

Text S1: A consensus picture of mitotic exit in budding yeast

Eukaryotic cells replicate and partition their DNA molecules in two distinct, coordinated phases. During S phase, each double-stranded DNA molecule is replicated to form two identical sister chromatids that are held together by cohesins (tethering proteins). During M phase, the cell builds a bipolar mitotic spindle, condenses its replicated chromosomes and aligns them on the metaphase plate with one chromatid attached to one pole of the spindle and its sister attached to the other pole, with the cohesin proteins maintaining tension between the two. When the replicated chromosomes are all properly aligned on the mitotic spindle (metaphase), the cell activates a protease (separase) that cleaves the cohesins, thereby allowing the sister chromatids to be pulled to opposite poles of the spindle (anaphase). Subsequently, in telophase, the cell divides into two daughter cells, each one containing a complete set of chromosomes. In a typical eukaryotic cell cycle, S and M phases are separated by two gaps, G1 and G2 phases, characterized (respectively) by unreplicated and replicated chromosomes.

Cyclin-dependent kinases (Cdks) are the master regulators of the eukaryotic cell cycle. When associated with the appropriate cyclins, Cdks phosphorylate specific target proteins and trigger crucial events of the cell cycle, including DNA replication, nuclear envelope breakdown, chromosome condensation, and spindle assembly. Entry into mitosis in the budding yeast cell is promoted by Cdk in complex with mitotic cyclins (Clb1, Clb2, Clb3, and Clb4) [1].

Cdk subunits are present in excess during the cell cycle. The activity of Cdk-cyclin complexes is regulated in three different ways [2]. First, the availability of cyclin subunits is controlled by transcription factors and by ubiquitin-dependent proteolysis systems. The anaphase-promoting complex (APC) [3], a multisubunit ubiquitin ligase, degrades cyclin proteins in anaphase. Second, Cdk/cyclin activity can be regulated by phosphorylation of the kinase subunit, and third, Cdk-cyclin dimers can be inactivated by binding to Cdk inhibitors (CKIs) [4-6]. The availability of a CKI depends on its production rate, which is governed by transcription factors, and its destruction rate, which is governed by a ubiquitin-ligase pathway [7].

Polo kinases (Plks) are also pivotal regulators of the M phase and are conserved from yeasts to humans. Plks play key roles during entry into mitosis, bipolar spindle formation, chromosome segregation, and cytokinesis [8]. In budding yeast, polo kinase (Cdc5) promotes anaphase by phosphorylating the cohesin subunit, Scc1, which enhances the cleavage of Scc1 by separase (Esp1) [9]. Cdc5 is required for proper spindle microtubule dynamics [10]. Cdc5 also influences Cdk activity by antagonizing Swe1, an inhibitory kinase for Cdk [11].

Cdc5 activity is regulated by transcriptional control, phosphorylation, and proteolysis. Cdk is important in maintaining Cdc5 activity *in vivo* and can stimulate Cdc5 activity through phosphorylation within a short region of the catalytic domain (T-loop) [12]. Cdc5 degradation involves the Cdh1/APC complex and depends on an amino-terminal destruction signal [13]. If Polo activity is compromised, as in *cdc5-1* mutant cells, the division-promoting proteins Cdc15, Dbf2, and Mob1 fail to be recruited to the spindle pole.

During prometaphase, when Cdk and Polo activities are high and replicated chromosomes are being aligned on the mitotic spindle, the spindle assembly checkpoint (SAC) holds Cdc20/APC in an inactive complex with Mad2, an essential SAC protein. When the chromosomes are all properly aligned, Mad2 disengages and Cdc20/APC initiates proteolysis of Pds1 (securin) [14], thereby liberating the protease Esp1 (separase), allowing it to cleave cohesins that hold sister chromatids together. In anaphase, sister chromatids are pulled apart by the elongating spindle [15]. Cdc20/APC also initiates partial degradation of mitotic cyclins.

To exit from mitosis and divide, a cell must reverse the phosphorylations that were carried out by Cdk as the cell entered mitosis [16]. In budding yeast, this reversal is normally accomplished in two stages: first, Cdk activity is reduced by partial degradation of Clb’s by Cdc20/APC [17], and second, the activation of a Cdk-opposing phosphatase, Cdc14, removes the phosphate groups from Cdk-target proteins. One of these target proteins is Cdh1, a homolog of Cdc20, which combines with the APC to promote complete degradation of Clb subunits [18,19]. Cdc14 also dephosphorylates and stabilizes Sic1 [20], which binds to any remaining Cdk/Clb dimers to form inactive trimeric complexes. Notice that the timing of events during mitotic exit (ME) is determined by the sequence of activation of these exit proteins. First, Cdc20 is activated, leading

to activation of separase and separation of sister chromatids (anaphase) and transient activation of Cdc14. Only after the sister chromatids have been separated does Cdc14 activate Cdh1 and Sic1, leading to the complete elimination of Cdk activity in telophase, accompanied by cell division and the return of mother and daughter cells to G1 phase.

The transient activation of Cdc14 during ME is a complex process. Prior to the metaphase-anaphase transition, Cdc14 is confined to the nucleolus in association with Net1, forming a complex called the regulator of nucleolar silencing and telophase exit (RENT) [21,22]. In anaphase and telophase, Cdc14 is released from the nucleolus into the nucleoplasm and cytoplasm, a process driven by the phosphorylation of Net1 by Cdk, Cdc5, and other mitotic-exit kinases [23-25]. Phosphorylated Net1 has a much lower affinity for Cdc14 and cannot sequester it in the nucleolus [23-25]. Net1 phosphorylation and Cdc14 release, in budding yeast, occur by two distinct regulatory pathways. At the onset of anaphase, Cdc14 dissociates from Net1 by the action of the Cdc14 early anaphase release (FEAR) pathway [26]. As anaphase proceeds, FEAR-induced Cdc14 activates a second pathway, the mitotic exit network (MEN), which sustains the release of Cdc14 and allows budding yeast cells to complete the transition to G1 phase [27-30]. If the MEN is blocked, the FEAR-released Cdc14 returns to the nucleolus before ME can be completed [26].

Components of the FEAR network include Esp1, Slk19, Spo12, Fob1, Cdk/Clb1,2, Cdc5, protein phosphatase 2A (PP2A) and its regulatory subunit, Cdc55 [26,30]. In prometaphase and metaphase, when Cdk and Cdc5 activities are high, PP2A/Cdc55 prevents premature Cdc14 release by rapidly dephosphorylating Net1. At anaphase onset, FEAR promotes the first wave of Net1 phosphorylation by downregulating the activity of PP2A/Cdc55 [31]. FEAR-released Cdc14 induces anaphase events, in particular nuclear movement into the bud compartment [23,32-36]. Full activation of Cdc14 by MEN is required to complete the process of ME [30].

MEN is an integral part of the spindle position checkpoint, a signal transduction system that monitors the spatial orientation of the anaphase spindle relative to the polarity axis of the cell at the bud neck [37]. If the spindle is properly positioned, then in late anaphase the daughter cell nucleus will be pushed into the bud compartment where its spindle pole body (SPB) will interact

with components of the bud cortex to activate MEN, to sustain the release of Cdc14, and to complete the events of telophase [37]. If the spindle is improperly positioned, the MEN will not be activated, Cdc14 will return prematurely to the nucleolus, and the cell will not divide, allowing time to reposition the spindle if possible [38].

The MEN pathway is a G-protein signaling cascade, composed of a GTPase (Tem1), a GTPase-activating protein (GAP; Bub2/Bfa1 complex), which converts active $Tem1^{GTP}$ into inactive $Tem1^{GDP}$, and a putative guanine nucleotide exchange factor (GEF; Lte1), which converts $Tem1^{GDP}$ into $Tem1^{GTP}$. $Tem1^{GTP}$, in combination with Cdc15, activates the Dbf2/Mob1 complex, a protein kinase that is responsible for the phosphorylation of Net1. Cdc5 also plays essential roles in MEN, including phosphorylation and inactivation of Bub2-Bfa1.

ME should occur only after successful segregation of the anaphase nuclei into the mother and daughter cell compartments. To enforce this requirement, Tem1 is kept inactive by the Bub2/Bfa1 complex. It appears that Tem1 is activated following the spindle elongation and successful segregation of the SPB into the daughter cell, where Tem1 encounters its putative exchange factor Lte1, which is concentrated in the bud cortex [39-41]. Lte1 and Bfa1 are also phosphorylated in a *CDC5*-dependent manner [29,42], suggesting that Cdc5 promotes Tem1 signaling by activating Lte1 and inhibiting Bub2/Bfa1. The dual pathways (FEAR and MEN) controlling ME in budding yeast have been called ‘double clutch’ control by [43].

References

1. Bloom J, Cross FR (2007) Multiple levels of cyclin specificity in cell-cycle control. Nat Rev Mol Cell Biol 8: 149-160.
2. DO M (2006) The Cell Cycle: Principles of Control. London: New Science Press.
3. Peters JM (2006) The anaphase promoting complex/cyclosome: a machine designed to destroy. Nat Rev Mol Cell Biol 7: 644-656.
4. Donovan JD, Toyn JH, Johnson AL, Johnston LH (1994) P40SDB25, a putative CDK inhibitor, has a role in the M/G1 transition in Saccharomyces cerevisiae. Genes & Development 8: 1640-1653.
5. Moreno S, Nurse P (1994) Regulation of progression through the G1 phase of the cell cycle by the rum1+ gene. Nature 367: 236-242.
6. Schwob E, B”hm T, Mendenhall MD, Nasmyth K (1994) The B-type cyclin kinase inhibitor p40SIC1 controls the G1 to S transition in S. cerevisiae. Cell 79: 233-244.

7. Benito J, Martin-Castellanos C, Moreno S (1998) Regulation of the G1 phase of the cell cycle by periodic stabilization and degradation of the p25rum1 CDK inhibitor. EMBO J 17: 482-497.
8. Archambault V, Glover DM (2009) Polo-like kinases: conservation and divergence in their functions and regulation. Nat Rev Mol Cell Biol 10: 265-275.
9. Alexandru G, Uhlmann F, Mechtler K, Poupart MA, Nasmyth K (2001) Phosphorylation of the cohesin subunit Scc1 by Polo/Cdc5 kinase regulates sister chromatid separation in yeast. Cell 105: 459-472.
10. Park CJ, Park JE, Karpova TS, Soung NK, Yu LR, et al. (2008) Requirement for the budding yeast polo kinase Cdc5 in proper microtubule growth and dynamics. Eukaryot Cell 7: 444-453.
11. Liang F, Jin F, Liu H, Wang Y (2009) The molecular function of the yeast polo-like kinase Cdc5 in Cdc14 release during early anaphase. Mol Biol Cell 20: 3671-3679.
12. Mortensen EM, Haas W, Gygi M, Gygi SP, Kellogg DR (2005) Cdc28-dependent regulation of the Cdc5/Polo kinase. Curr Biol 15: 2033-2037.
13. Charles JF, Jaspersen SL, Tinker-Kulberg RL, Hwang L, Szidon A, et al. (1998) The Polo-related kinase Cdc5 activates and is destroyed by the mitotic cyclin destruction machinery in S. cerevisiae. Curr Biol 8: 497-507.
14. Zachariae W, Nasmyth K (1999) Whose end is destruction: cell division and the anaphase-promoting complex. Genes Dev 13: 2039-2058.
15. Nasmyth K, Peters J-M, Uhlmann F (2000) Splitting the Chromosome: Cutting the Ties That Bind Sister Chromatids. Science 288: 1379-1384.
16. Jin F, Liu H, Liang F, Rizkallah R, Hurt MM, et al. (2008) Temporal control of the dephosphorylation of Cdk substrates by mitotic exit pathways in budding yeast. Proc Natl Acad Sci U S A 105: 16177-16182.
17. Cerutti L, Simanis V (2000) Controlling the end of the cell cycle. Curr Opin Genet Dev 10: 65-69.
18. Schwab M, Lutum AS, Seufert W (1997) Yeast Hct1 is a regulator of Clb2 cyclin proteolysis. Cell 90: 683-693.
19. Visintin R, Prinz S, Amon A (1997) CDC20 and CDH1: a family of substrate-specific activators of APC-dependent proteolysis. Science 278: 460-463.
20. Visintin R, Craig K, Hwang ES, Prinz S, Tyers M, et al. (1998) The phosphatase Cdc14 triggers mitotic exit by reversal of Cdk-dependent phosphorylation. Mol Cell 2: 709-718.
21. Shou W, Seol JH, Shevchenko A, Baskerville C, Moazed D, et al. (1999) Exit from mitosis is triggered by Tem1-dependent release of the protein phosphatase Cdc14 from nucleolar RENT complex. Cell 97: 233-244.
22. Visintin R, Hwang ES, Amon A (1999) Cfi1 prevents premature exit from mitosis by anchoring Cdc14 phosphatase in the nucleolus. Nature 398: 818-823.
23. Azzam R, Chen SL, Shou W, Mah AS, Alexandru G, et al. (2004) Phosphorylation by Cyclin B-Cdk Underlies Release of Mitotic Exit Activator Cdc14 from the Nucleolus. Science 305: 516-519.
24. Shou W, Azzam R, Chen S, Huddleston M, Baskerville C, et al. (2002) Cdc5 influences phosphorylation of Net1 and disassembly of the RENT complex. BMC Molecular Biology 3: 3-3.
25. Yoshida S, Asakawa K, Toh-e A (2002) Mitotic exit network controls the localization of Cdc14 to the spindle pole body in Saccharomyces cerevisiae. Curr Biol 12: 944-950.

26. Stegmeier F, Visintin R, Amon A (2002) Separase, polo kinase, the kinetochore protein Slk19, and Spo12 function in a network that controls Cdc14 localization during early anaphase. Cell 108: 207-220.
27. Jaspersen SL, Charles JF, Tinker-Kulberg RL, Morgan DO (1998) A late mitotic regulatory network controlling cyclin destruction in Saccharomyces cerevisiae. Mol Biol Cell 9: 2803-2817.
28. Jaspersen SL, Morgan DO (2000) Cdc14 activates cdc15 to promote mitotic exit in budding yeast. Curr Biol 10: 615-618.
29. Lee SE, Jensen S, Frenz LM, Johnson AL, Fesquet D, et al. (2001) The Bub2-dependent mitotic pathway in yeast acts every cell cycle and regulates cytokinesis. J Cell Sci 114: 2345-2354.
30. Stegmeier F, Amon A (2004) Closing mitosis: the functions of the Cdc14 phosphatase and its regulation. Annu Rev Genet 38: 203-232.
31. Queralt E, Lehane C, Novak B, Uhlmann F (2006) Downregulation of PP2A(Cdc55) phosphatase by separase initiates mitotic exit in budding yeast. Cell 125: 719-732.
32. Ross KE, Cohen-Fix O (2004) A role for the FEAR pathway in nuclear positioning during anaphase. Dev Cell 6: 729-735.
33. Sullivan M, Higuchi T, Katis VL, Uhlmann F (2004) Cdc14 phosphatase induces rDNA condensation and resolves cohesin-independent cohesion during budding yeast anaphase. Cell 117: 471-482.
34. D'Amours D, Stegmeier F, Amon A (2004) Cdc14 and condensin control the dissolution of cohesin-independent chromosome linkages at repeated DNA. Cell 117: 455-469.
35. Higuchi T, Uhlmann F (2005) Stabilization of microtubule dynamics at anaphase onset promotes chromosome segregation. Nature 433: 171-176.
36. Lu Y, Cross F (2009) Mitotic exit in the absence of separase activity. Mol Biol Cell 20: 1576-1591.
37. Bosl WJ, Li R (2005) Mitotic-exit control as an evolved complex system. Cell 121: 325-333.
38. Geymonat M, Jensen S, Johnston LH (2002) Mitotic exit: the Cdc14 double cross. Curr Biol 12: R482-484.
39. Yeh E, Skibbens RV, Cheng JW, Salmon ED, Bloom K (1995) Spindle dynamics and cell cycle regulation of dynein in the budding yeast, Saccharomyces cerevisiae. J Cell Biol 130: 687-700.
40. Bardin AJ, Visintin R, Amon A (2000) A mechanism for coupling exit from mitosis to partitioning of the nucleus. Cell 102: 21-31.
41. Pereira G, Hofken T, Grindlay J, Manson C, Schiebel E (2000) The Bub2p spindle checkpoint links nuclear migration with mitotic exit. Mol Cell 6: 1-10.
42. Hu F, Wang Y, Liu D, Li Y, Qin J, et al. (2001) Regulation of the Bub2/Bfa1 GAP complex by Cdc5 and cell cycle checkpoints. Cell 107: 655-665.
43. D'Amours D, Amon A (2004) At the interface between signaling and executing anaphase--Cdc14 and the FEAR network. Genes Dev 18: 2581-2595.
