# Supplementary material for: A Mathematical Model of Mitotic Exit in Budding Yeast: The Role of Polo Kinase
Source: PLoS One. 2012 Feb 23;7(2):e30810. doi: 10.1371/journal.pone.0030810 (PMC3285609; doi:10.1371/journal.pone.0030810)
Supplement: Text S2 — Assumptions of the Model. (PDF) [file pone.0030810.s005.pdf]

Text S2: Assumptions of the model

A common trend in developing complex models in molecular cell biology is to start from a simple model and then refine and expand it step by step (as more data become available) into an increasingly more comprehensive model. We have taken this approach in our study of mitotic exit in budding yeast cell cycle. We have limited the scope of our model so that it can be based largely on experimental observations, is not overwhelmed with assumptions, and is able to make predictions. Obviously, at any stage of modeling there will be facts that have not yet been incorporated and thus are out of the scope of the model. Major assumptions made in the process of building our model are listed below. Some technical assumptions of the mathematical model are described also in Supplementary Table S1.

1. We model average behavior of cells and do not address naturally occurring, stochastic fluctuations in cell cycle progression.
2. ODEs are given to describe the active forms (phosphorylated Cdc5, dephosphorylated Cdc15, Cdh1, and Net1) of the proteins for those who have both active and inactive form. The corresponding inactive form is given by: [total]-[active].
3. All synthesis, degradation, binding and dissociation reactions are described by mass action law. Rate constants starting with *k* have a dimension of min^{-1}, and other parameters are dimensionless.
4. Activation and inactivation of Tem1, reversible phosphorylation and dephosphorylation reactions of Cdc5, Cdh1, Net1, and Cdc15 are described by Michaelis-Menten (MM) kinetics.
5. Cdc28 and APC are neglected in the equations because they are constant. Clb2 represents Cd28/Clb2 complexes and Cdk represents the kinase activity of Cdc28-Clb2 dimers; in the absence of chemical inhibitor (INH) Clb2 represents the kinase activity. Similarly, Cdc20 and Cdh1 represent the concentration of active APC/Cdc20 and APC/Cdh1 complexes. Clb2 is synthesized at constant rate, and degraded by Cdh1 and Cdc20 [1-3].
6. The total amount of Cdc14 is observed to be constant through the cell cycle. Cdc14 contributes to Net1 dephosphorylation [4,5].
7. Cdc20 is synthesized at constant rate and degraded by Cdh1 [6-9].

8. Cdh1 is considered to be constant [7]. Cdc14 promotes Cdh1 activation by dephosphorylation, and Clb2 promotes its inactivation by phoshorylation [10-12]. Pds1 is synthesized at constant rate, and degraded by Cdc20 [13].
9. Esp1 is synthesized at constant rate and degraded with mass action kinetics. Esp1 forms complex with Pds1. In the complex, both Esp1 and Pds1 can be degraded.
10. Net1 is constant during the cell cycle. It is phosphorylated by Cdk/Clb2 [14], Polo kinase (Cdc5) [15,16], and active MEN (ACTMEN) [5,16-19], and dephosphorylated by Cdc14 and PP2A/Cdc55 [4,5]. We assume that Clb2, Cdc5, and ACTMEN phosphorylate Net1 both free and bound to Cdc14 in RENT, RENTP, and PRENT. Phosphorylated PRENT and PRENTP complexes dissociate quickly into PNet1, PNet1P, and Cdc14 whereas RENTP is a stable complex similar to RENT.
11. PP2A/Cdc55 is inhibited by Esp1 as (1+kpp*ki*Esp1)/(1+ki*Esp1).
12. S is a variable representing spindle elongation by the equation, S=exp((-ks)*(t-tm))*(1.0-*N*)*C*(1.0-*SCRRDD*). Parameter "*C*" represents cohesion cleavage. "*N*" is defined to simulate experiments with the drug nocadozole. In nocadozole, parameter *N* becomes 1, and consequently *S* becomes zero which means no spindle elongation. Similarly, *SCRRDD* is used to simulate "*GAL-SCC1-RRDD*" mutants. When cohesion is nondegradable, *SCRRDD* is set to 1.
13. Polo activity does not change quantitatively during mitosis [20]. Cdc5 is made at constant rate, and degraded by Cdh1 [21] with mass action kinetics. Cdc5 is activated by Cdk [22], and inactivated by a phosphatase with Michaelis-Menten kinetics.
14. "ACT" is used to represent the active form of the species. For example: in equation "ACTMEN=effc15*MEN", the parameter "effc15" quantifies the efficiency of the variable "MEN". When effc15 equals to zero, MEN becomes inactive, and when effc15 equals to 1, then MEN is fully active.
15. Tem1 concentration is constant. To simplify quantitative description, Tem1 is activated by Polo kinase and spindle elongation (represented by S), and inactivated by PP2A/Cdc55 and Cdc14 rather than acting through GAP and GEF. Tem1 has 3 forms, GDP bound form (TEM1i, inactive), GTP-bound free form (TEM1a), and a complex of Tem1-GTP and non-phosphorylated Cdc15, which is the active MEN.
16. MEN becomes inactive and falls apart if the Tem1 becomes Tem1-GDP (by PP2A and

Cdc14) or if the Cdc15 becomes phosphorylated (by Cdk). Cdc15 concentration is constant. Similarly, Cdc15 has 3 forms, non-phosphorylated Cdc15 (Cdc15a), phosphorylated (Cdc15P, inactive) and in the MEN complex. Cdk is the kinase that phosphorylates Cdc15a and makes it inactive, and Cdc14 is the opposing phosphatase.

17. We recognize the importance of spatial controls in the cell cycle. However, at this stage, we are trying to model the mitotic exit as far as possible without explicitly tracking the spatial localization of regulatory proteins. That would require a more sophisticated mathematical framework and is planned for the next stage of the model. Right after metaphase and before chromosome segregation, we keep track of proteins in the cell (in nucleus, cytoplasm, and SPBs). So, initial conditions of the variables are obtained for metaphase-arrested cells (*cdc20Δ GAL-CDC20*). Then they are released from the block by inducing expression of *CDC20* with galactose.

Since the consensus picture of the regulation of most ME proteins (summarized in Supplementary Text S1) has not changed significantly since the publication of Queralt's model, many of our modeling assumptions are the same as or similar to the assumptions made by Queralt et al. [23].

References

1. Schwab M, Lutum AS, Seufert W (1997) Yeast Hct1 is a regulator of Clb2 cyclin proteolysis. Cell 90: 683-693.
2. Visintin R, Prinz S, Amon A (1997) CDC20 and CDH1: a family of substrate-specific activators of APC-dependent proteolysis. Science 278: 460-463.
3. Wasch R, Cross FR (2002) APC-dependent proteolysis of the mitotic cyclin Clb2 is essential for mitotic exit. Nature 418: 556-562.
4. Shou W, Seol JH, Shevchenko A, Baskerville C, Moazed D, et al. (1999) Exit from mitosis is triggered by Tem1-dependent release of the protein phosphatase Cdc14 from nucleolar RENT complex. Cell 97: 233-244.
5. Jaspersen SL, Morgan DO (2000) Cdc14 activates cdc15 to promote mitotic exit in budding yeast. Curr Biol 10: 615-618.
6. Shirayama M, Zachariae W, Ciosk R, Nasmyth K (1998) The Polo-like kinase Cdc5p and the WD-repeat protein Cdc20p/fizzy are regulators and substrates of the anaphase promoting complex in Saccharomyces cerevisiae. The EMBO Journal 17: 1336?1349-1336?1349.
7. Prinz S, Hwang ES, Visintin R, Amon A (1998) The regulation of Cdc20 proteolysis reveals a role for APC components Cdc23 and Cdc27 during S phase and early mitosis. Curr Biol 8: 750-760.

8. Fang G, Yu H, Kirschner MW (1998) Direct binding of CDC20 protein family members activates the anaphase-promoting complex in mitosis and G1. Mol Cell 2: 163-171.
9. Pfleger CM, Kirschner MW (2000) The KEN box: an APC recognition signal distinct from the D box targeted by Cdh1. Genes Dev 14: 655-665.
10. Visintin R, Craig K, Hwang ES, Prinz S, Tyers M, et al. (1998) The phosphatase Cdc14 triggers mitotic exit by reversal of Cdk-dependent phosphorylation. Mol Cell 2: 709-718.
11. Zachariae W, Schwab M, Nasmyth K, Seufert W (1998) Control of cyclin ubiquitination by CDK-regulated binding of Hct1 to the anaphase promoting complex. Science 282: 1721-1724.
12. Jaspersen SL, Charles JF, Morgan DO (1999) Inhibitory phosphorylation of the APC regulator Hct1 is controlled by the kinase Cdc28 and the phosphatase Cdc14. Curr Biol 9: 227-236.
13. Sethi N, Monteagudo MC, Koshland D, Hogan E, Burke DJ (1991) The CDC20 gene product of Saccharomyces cerevisiae, a beta-transducin homolog, is required for a subset of microtubule-dependent cellular processes. Mol Cell Biol 11: 5592-5602.
14. Azzam R, Chen SL, Shou W, Mah AS, Alexandru G, et al. (2004) Phosphorylation by Cyclin B-Cdk Underlies Release of Mitotic Exit Activator Cdc14 from the Nucleolus. Science 305: 516-519.
15. Shou W, Azzam R, Chen S, Huddleston M, Baskerville C, et al. (2002) Cdc5 influences phosphorylation of Net1 and disassembly of the RENT complex. BMC Molecular Biology 3: 3-3.
16. Yoshida S, Toh-e A (2002) Budding yeast Cdc5 phosphorylates Net1 and assists Cdc14 release from the nucleolus. Biochemical and Biophysical Research Communications 294: 687-691.
17. Jaspersen SL, Charles JF, Tinker-Kulberg RL, Morgan DO (1998) A late mitotic regulatory network controlling cyclin destruction in Saccharomyces cerevisiae. Mol Biol Cell 9: 2803-2817.
18. Lee SE, Jensen S, Frenz LM, Johnson AL, Fesquet D, et al. (2001) The Bub2-dependent mitotic pathway in yeast acts every cell cycle and regulates cytokinesis. J Cell Sci 114: 2345-2354.
19. Stegmeier F, Amon A (2004) Closing mitosis: the functions of the Cdc14 phosphatase and its regulation. Annu Rev Genet 38: 203-232.
20. Cheng L, Hunke L, Hardy CF (1998) Cell cycle regulation of the Saccharomyces cerevisiae polo-like kinase cdc5p. Mol Cell Biol 18: 7360-7370.
21. Charles JF, Jaspersen SL, Tinker-Kulberg RL, Hwang L, Szidon A, et al. (1998) The Polo-related kinase Cdc5 activates and is destroyed by the mitotic cyclin destruction machinery in S. cerevisiae. Curr Biol 8: 497-507.
22. Mortensen EM, Haas W, Gygi M, Gygi SP, Kellogg DR (2005) Cdc28-dependent regulation of the Cdc5/Polo kinase. Curr Biol 15: 2033-2037.
23. Queralt E, Lehane C, Novak B, Uhlmann F (2006) Downregulation of PP2A(Cdc55) phosphatase by separase initiates mitotic exit in budding yeast. Cell 125: 719-732.
